# Supplementary material for: Syncytin-mediated open-ended membrane tubular connections facilitate the intercellular transfer of cargos including Cas9 protein
Source: eLife. 2023 Mar 10;12:e84391. doi: 10.7554/eLife.84391 (PMC10112890; doi:10.7554/eLife.84391)
Supplement: Figure 1—source data 1. [file elife-84391-fig1-data1.zip › Figure 1-source data 1/Figure 1-source data 1.pdf]

Figure 1B

uncropped blots

Purified exosomes

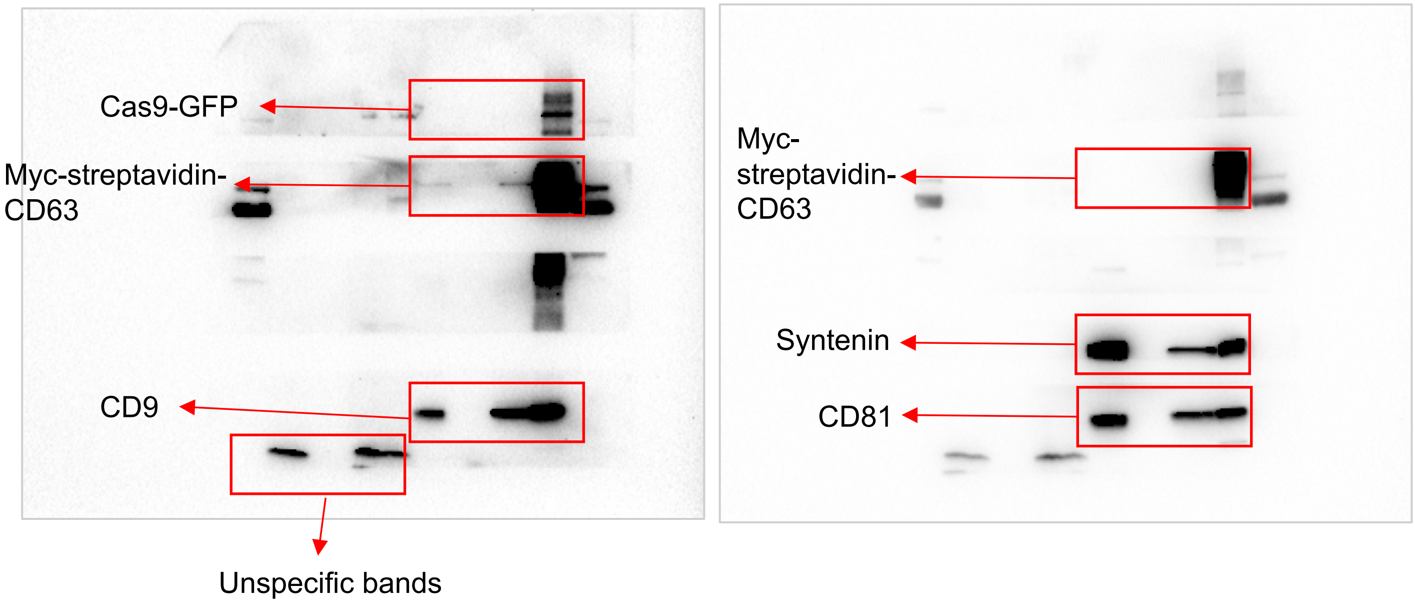

B

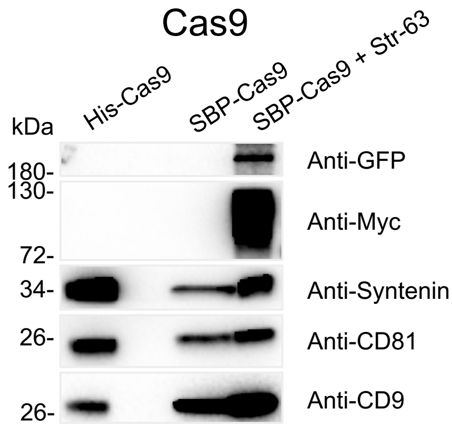

Cas9-GFP protein was detected in exosomes from the cells expressing both SBP-Flag-Cas9-GFP and Myc-Streptavidin-CD63-mCherry.
