# Supplementary figures and images for: Syncytin-mediated open-ended membrane tubular connections facilitate the intercellular transfer of cargos including Cas9 protein
Source: eLife. 2023 Mar 10;12:e84391. doi: 10.7554/eLife.84391 (PMC10112890; doi:10.7554/eLife.84391)

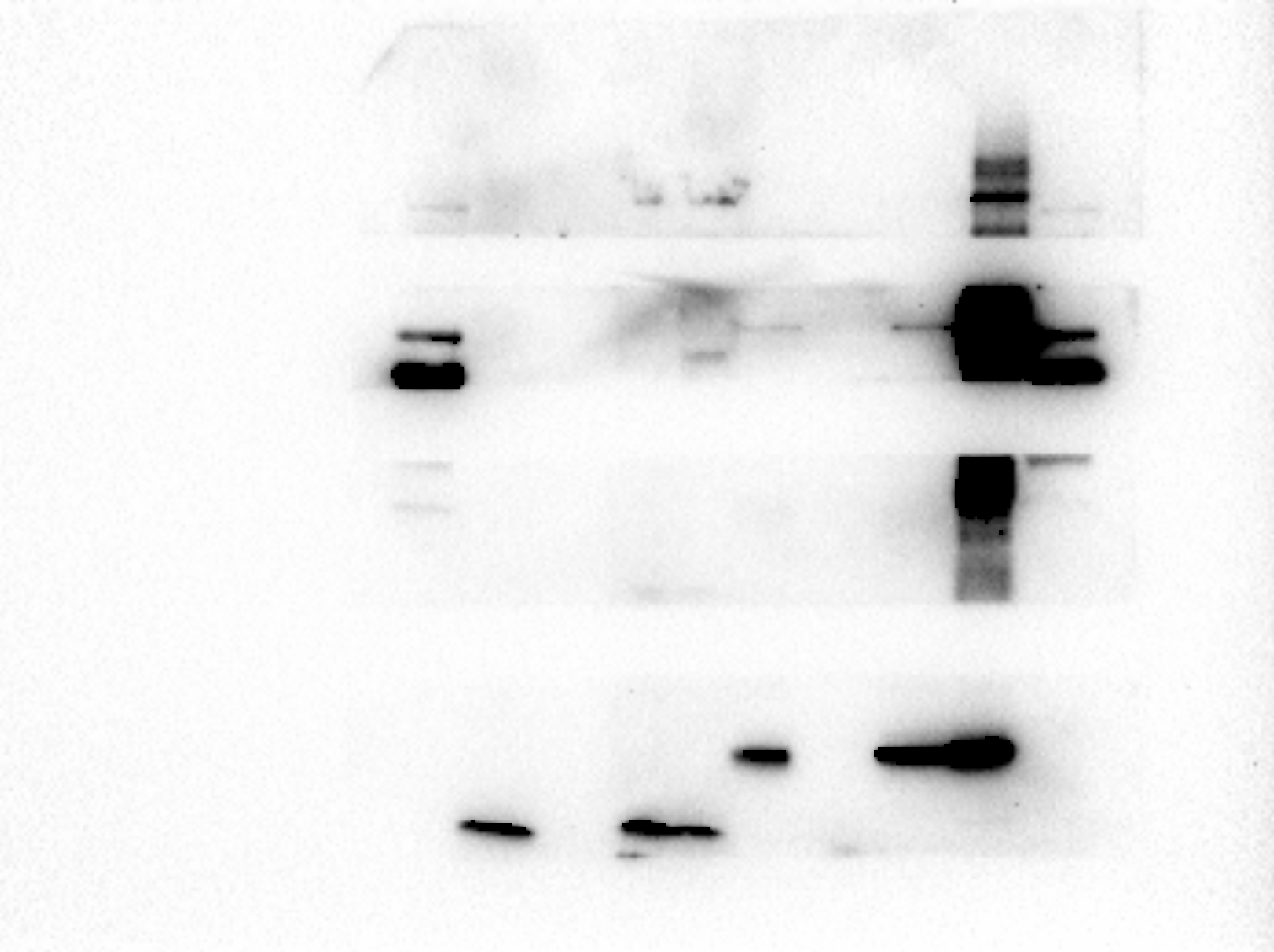

Supplement: Figure 1—source data 1. [file elife-84391-fig1-data1.zip › Figure 1-source data 1/Figure 1B-unmodified blot for anti-gfp anti-myc anti-CD63 anti-CD9 in exosomes.tif]

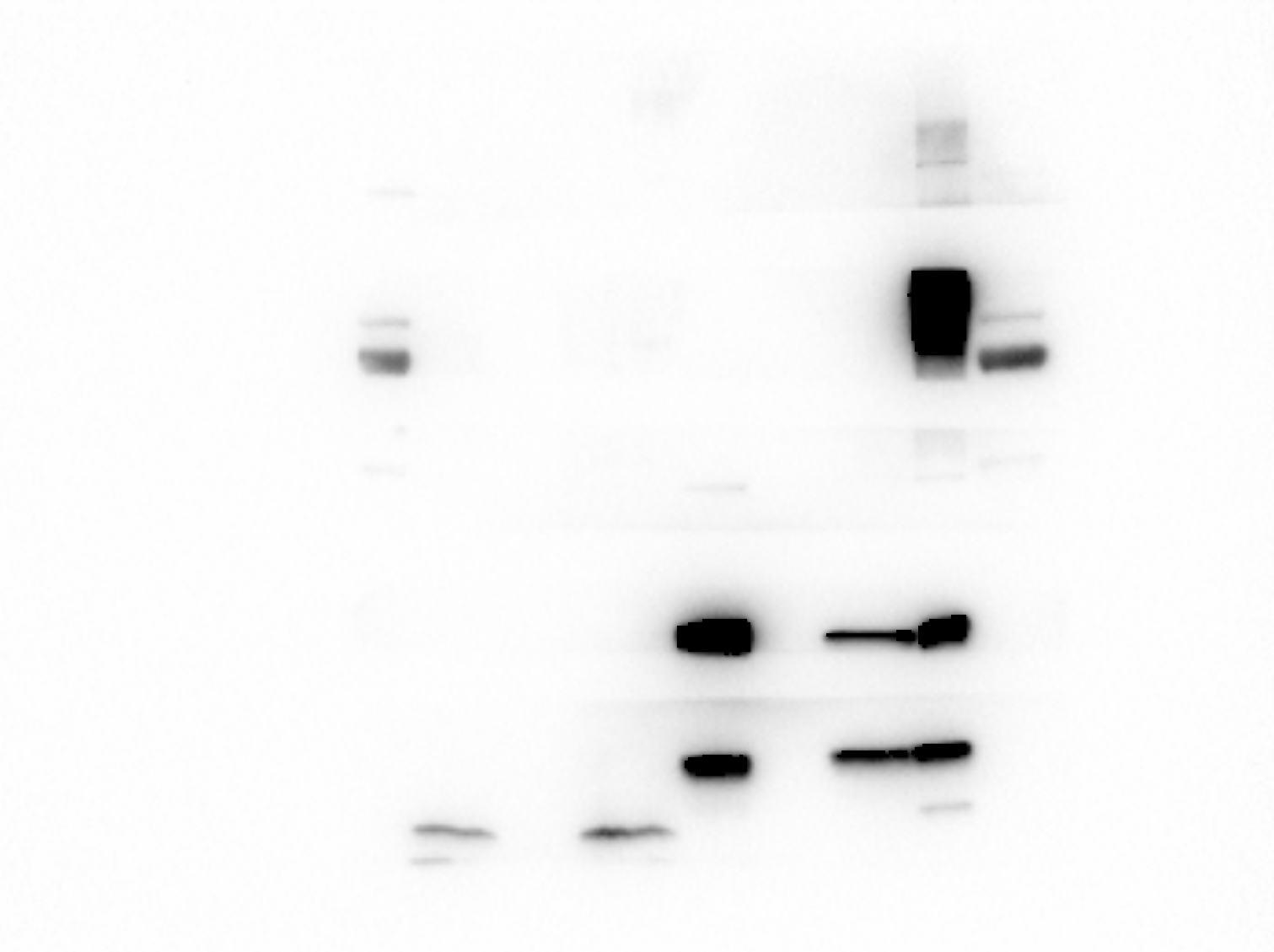

Supplement: Figure 1—source data 1. [file elife-84391-fig1-data1.zip › Figure 1-source data 1/Figure 1B-unmodified blot for anti-myc anti-syntenin anti-CD81 in exosomes.tif]

# Figure 1-figure supplement 1A

uncropped blots

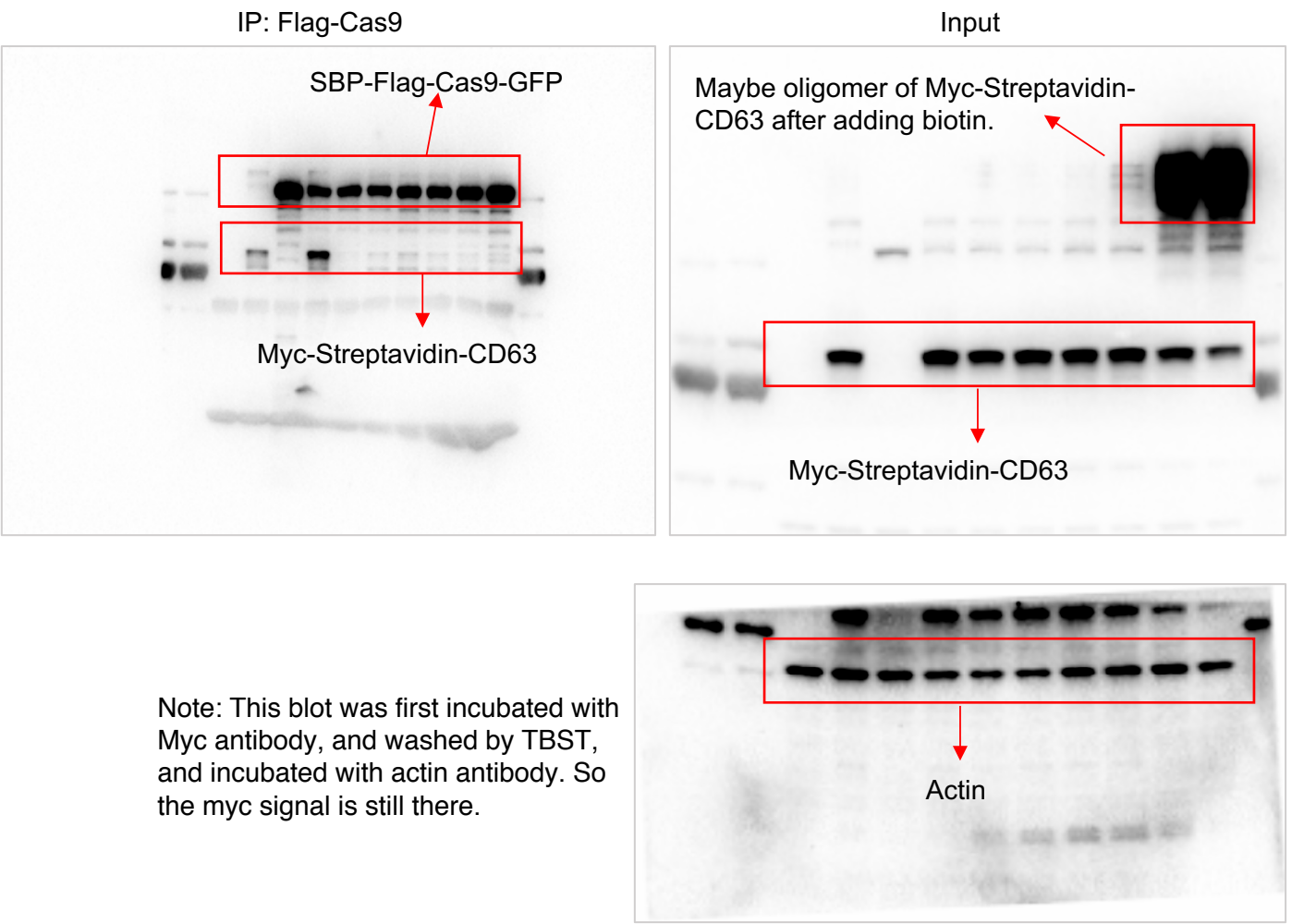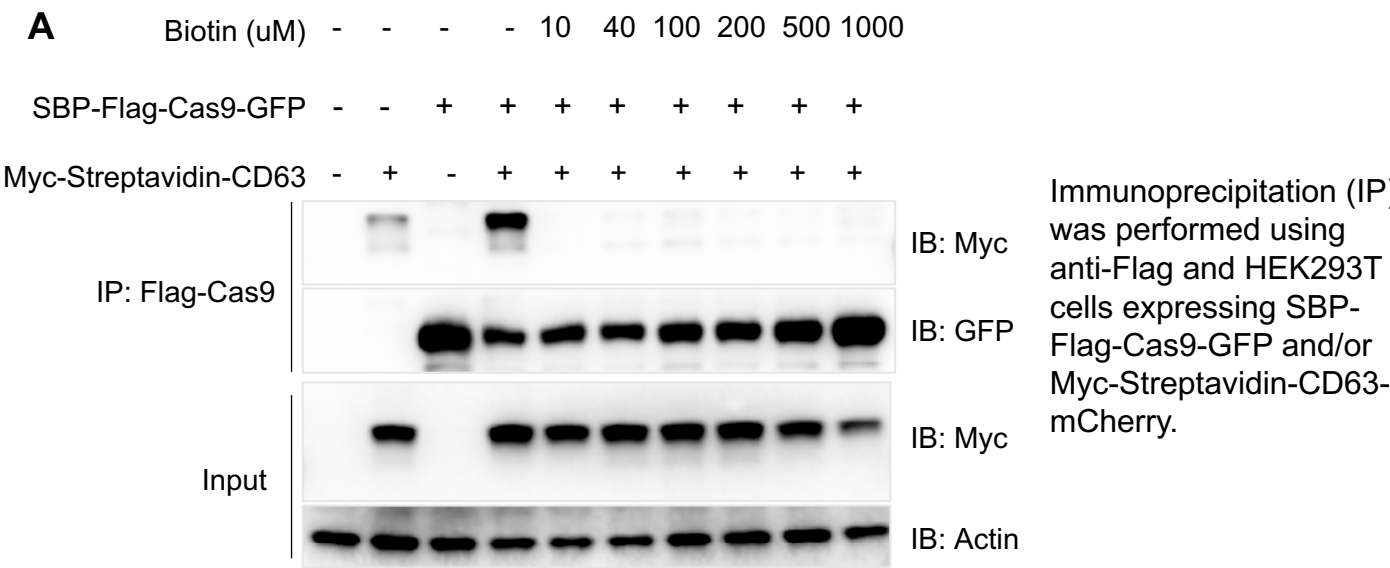

Supplement: Figure 1—figure supplement 1—source data 1. [file elife-84391-fig1-figsupp1-data1.zip › Figure 1-figure supplement 1-source data 1/Figure 1-figure supplement 1-source data 1.pdf]

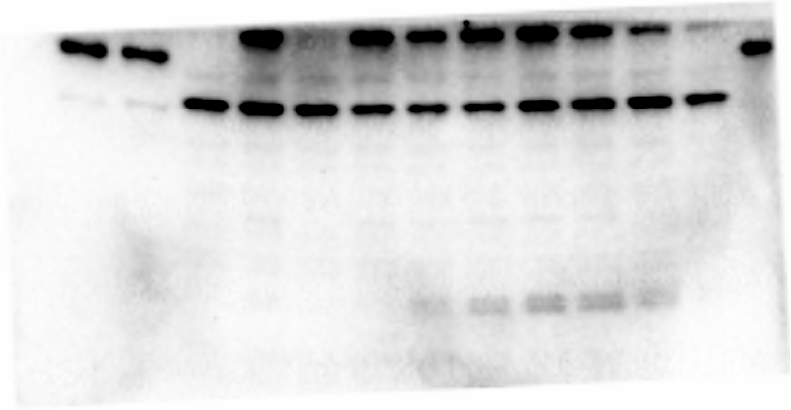

Supplement: Figure 1—figure supplement 1—source data 1. [file elife-84391-fig1-figsupp1-data1.zip › Figure 1-figure supplement 1-source data 1/Figure 1-figure supplement 1A-unmodified blot for anti-actin in input samples.tif]

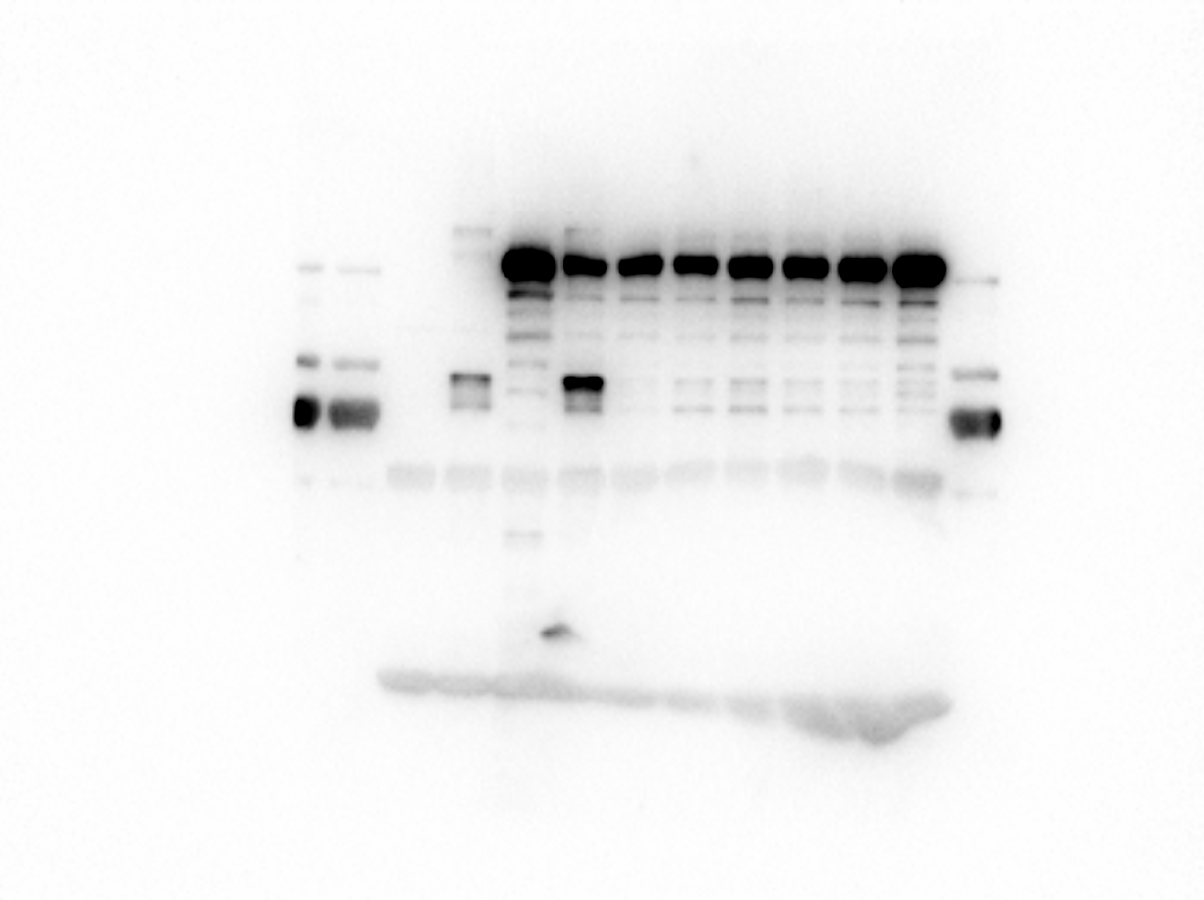

Supplement: Figure 1—figure supplement 1—source data 1. [file elife-84391-fig1-figsupp1-data1.zip › Figure 1-figure supplement 1-source data 1/Figure 1-figure supplement 1A-unmodified blot for anti-GFP and anti-myc in IP samples.tif]

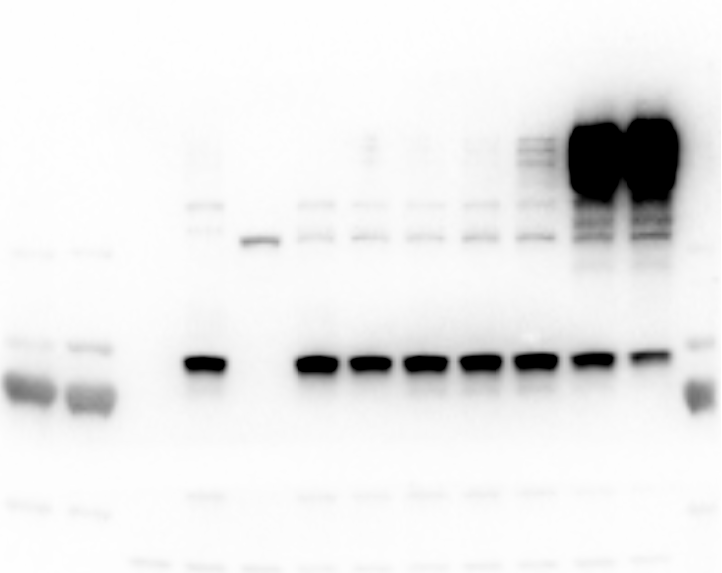

Supplement: Figure 1—figure supplement 1—source data 1. [file elife-84391-fig1-figsupp1-data1.zip › Figure 1-figure supplement 1-source data 1/Figure 1-figure supplement 1A-unmodified blot for anti-myc in input samples.tif]

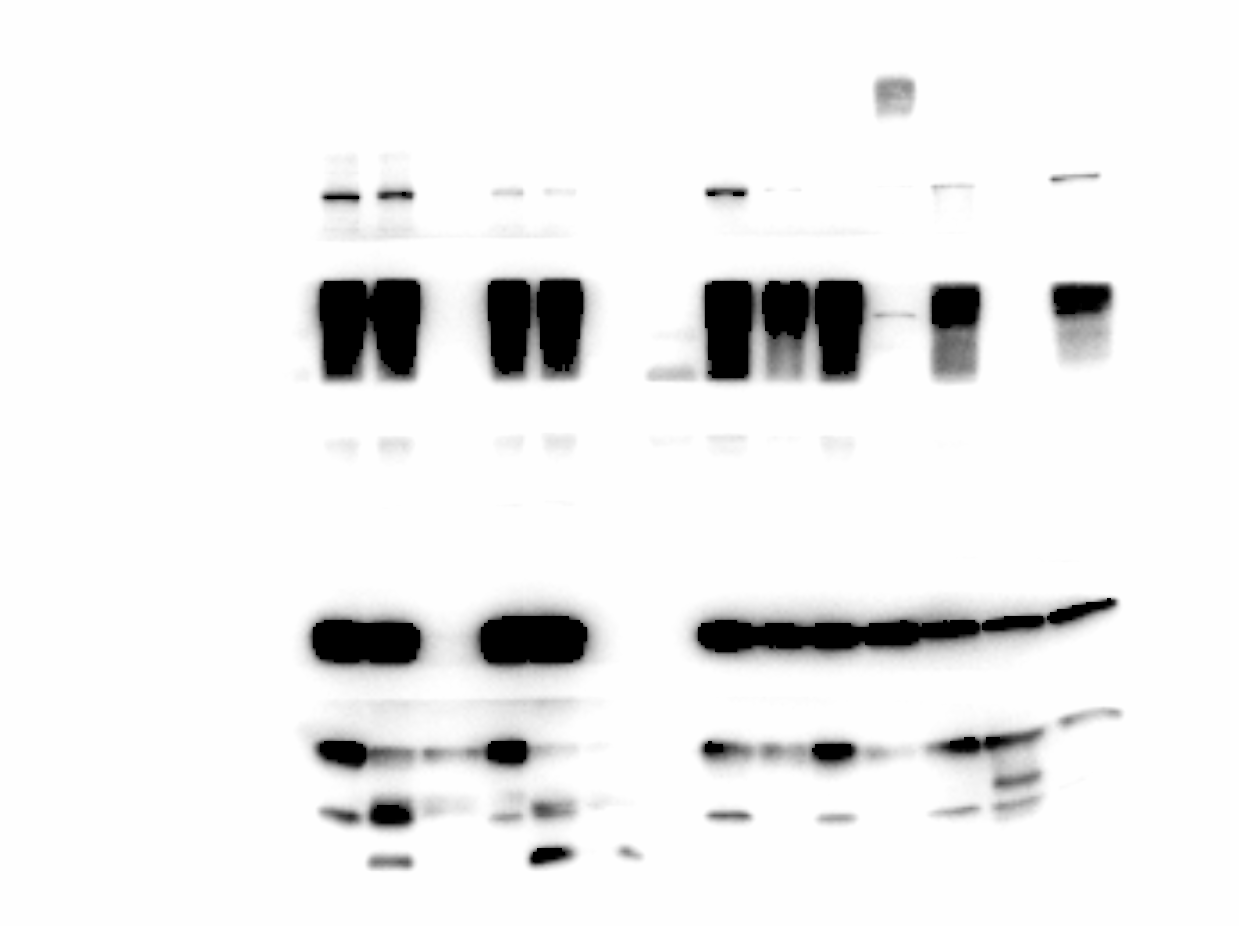

Supplement: Figure 1—figure supplement 1—source data 2. [file elife-84391-fig1-figsupp1-data2.zip › Figure 1-figure supplement 1-source data 2/Figure 1-figure supplement 1B-unmodified blot for anti-GFP anti-myc anti-TSG101 anti-syntenin anti-CD81.tif]

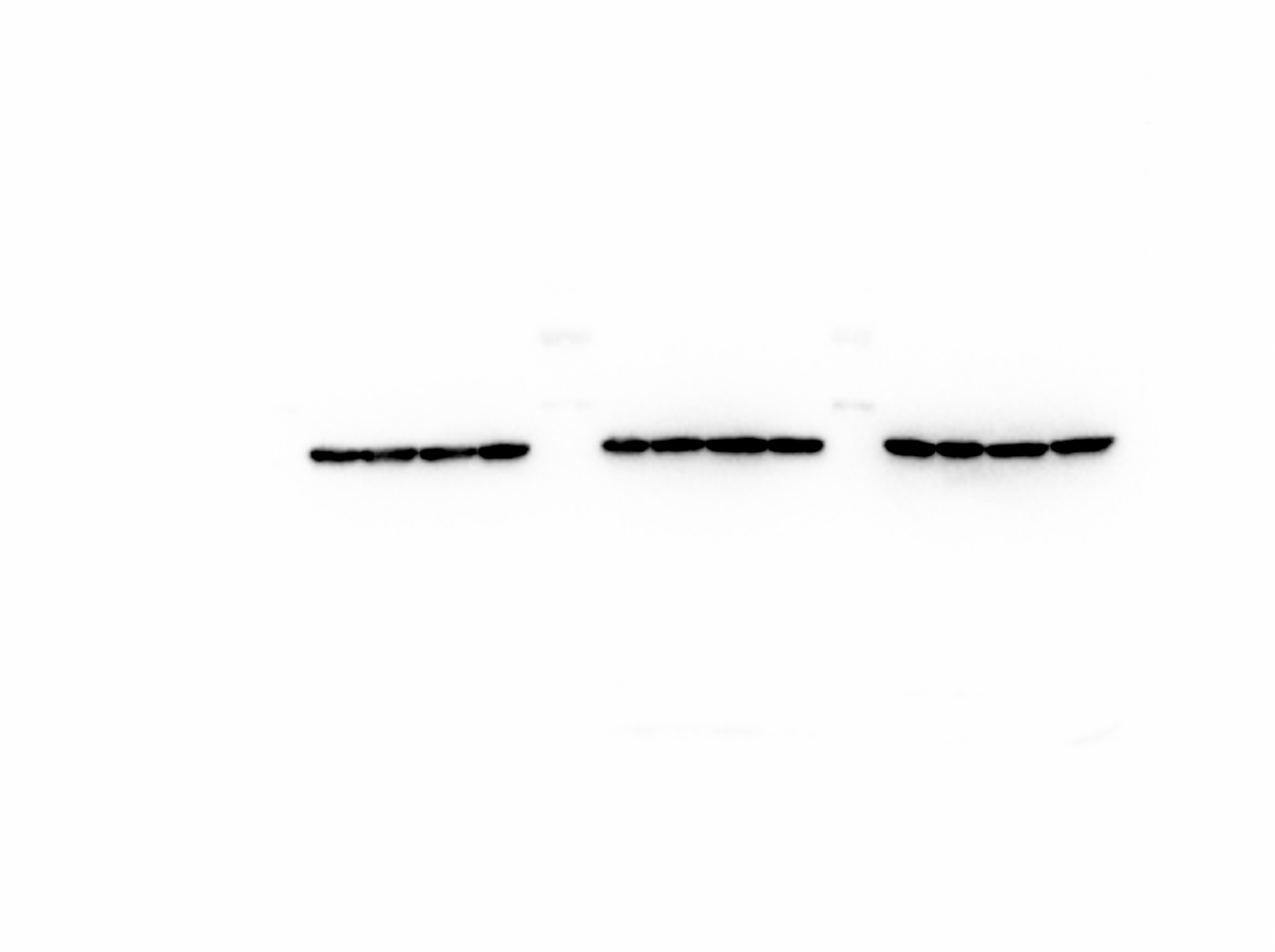

Supplement: Figure 3—figure supplement 2—source data 1. [file elife-84391-fig3-figsupp2-data1.zip › Figure 3-figure supplement 2-source data 1/Figure 3-figure supplement 2A-unmodified blot for anti-actin.tif]

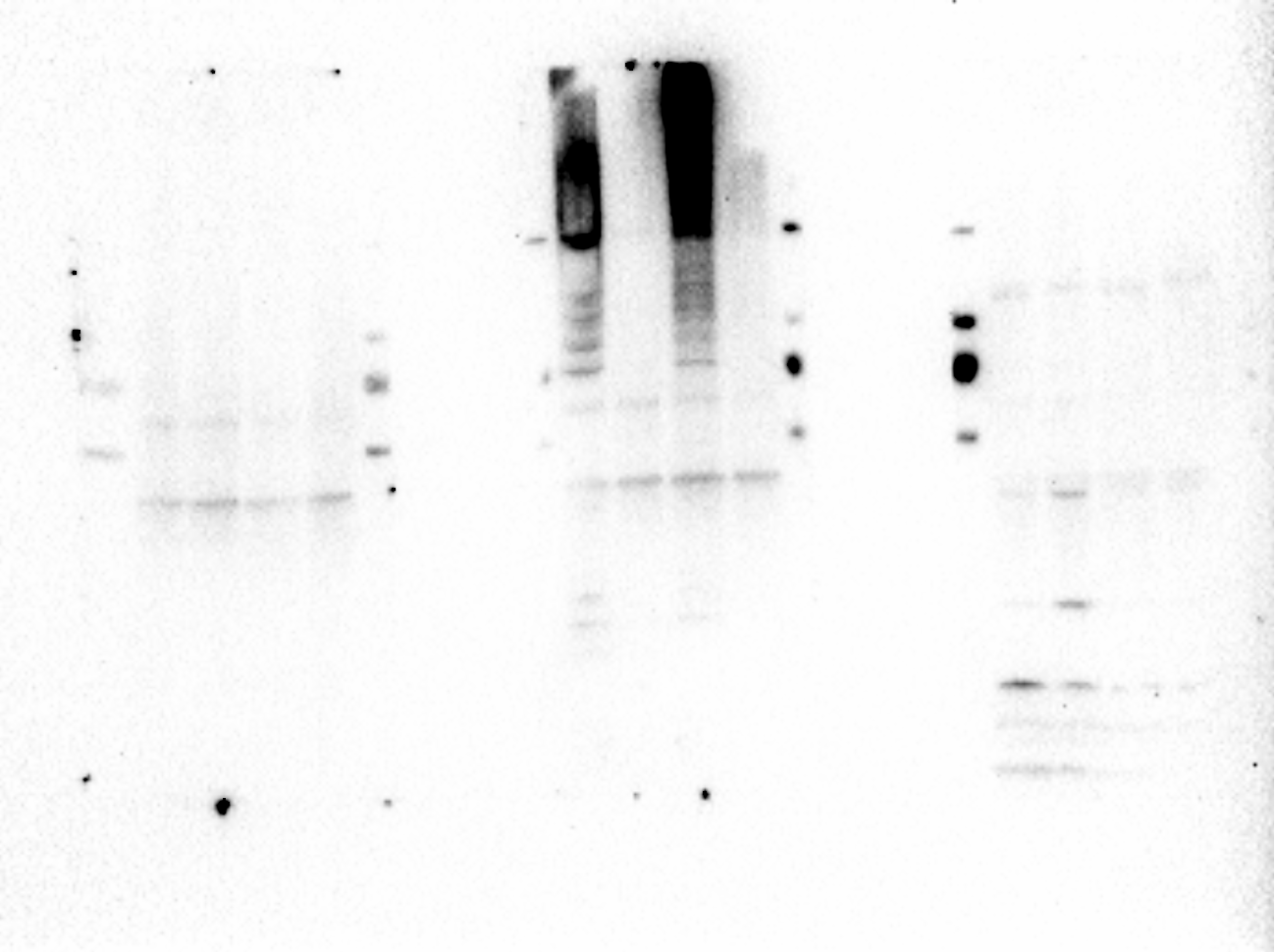

Supplement: Figure 3—figure supplement 2—source data 1. [file elife-84391-fig3-figsupp2-data1.zip › Figure 3-figure supplement 2-source data 1/Figure 3-figure supplement 2A-unmodified blot for anti-CLTC.tif]

Figure 3-figure supplement 2B

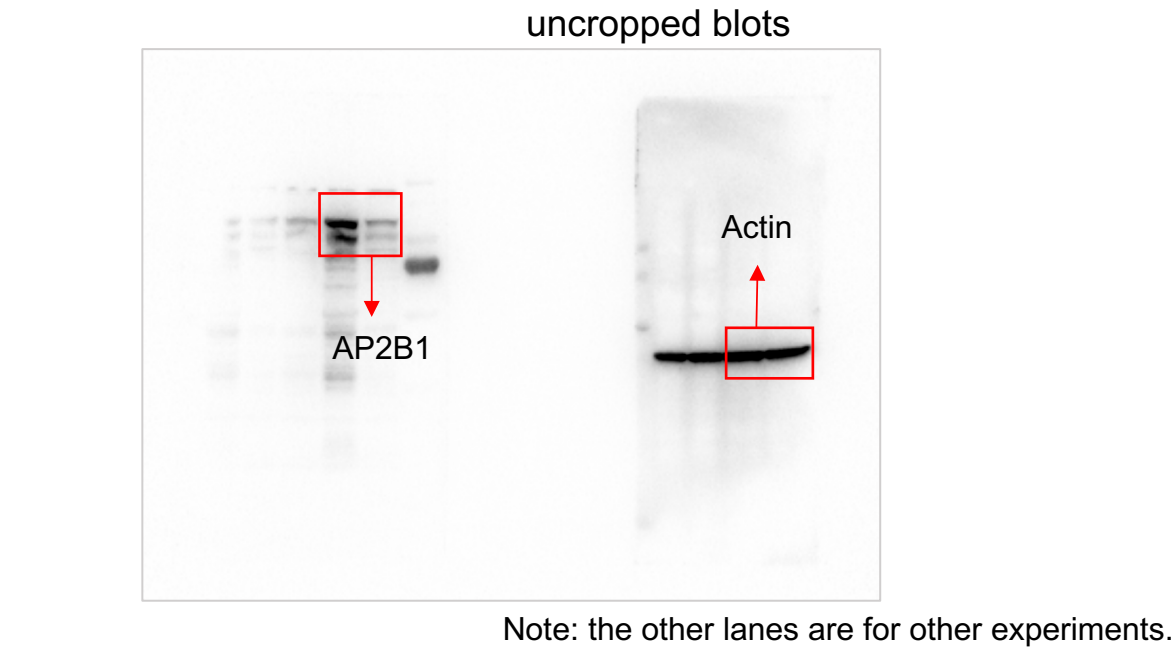

**B**

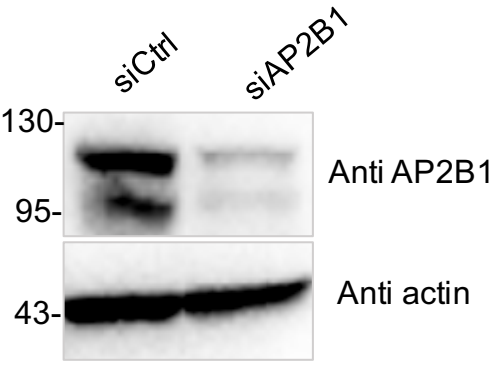

AP2B1 was knocked-down by siRNA in MDA-MB-231 cells.

Supplement: Figure 3—figure supplement 2—source data 2. [file elife-84391-fig3-figsupp2-data2.zip › Figure 3-figure supplement 2-source data 2/Figure 3-figure supplement 2-source data 2.pdf]

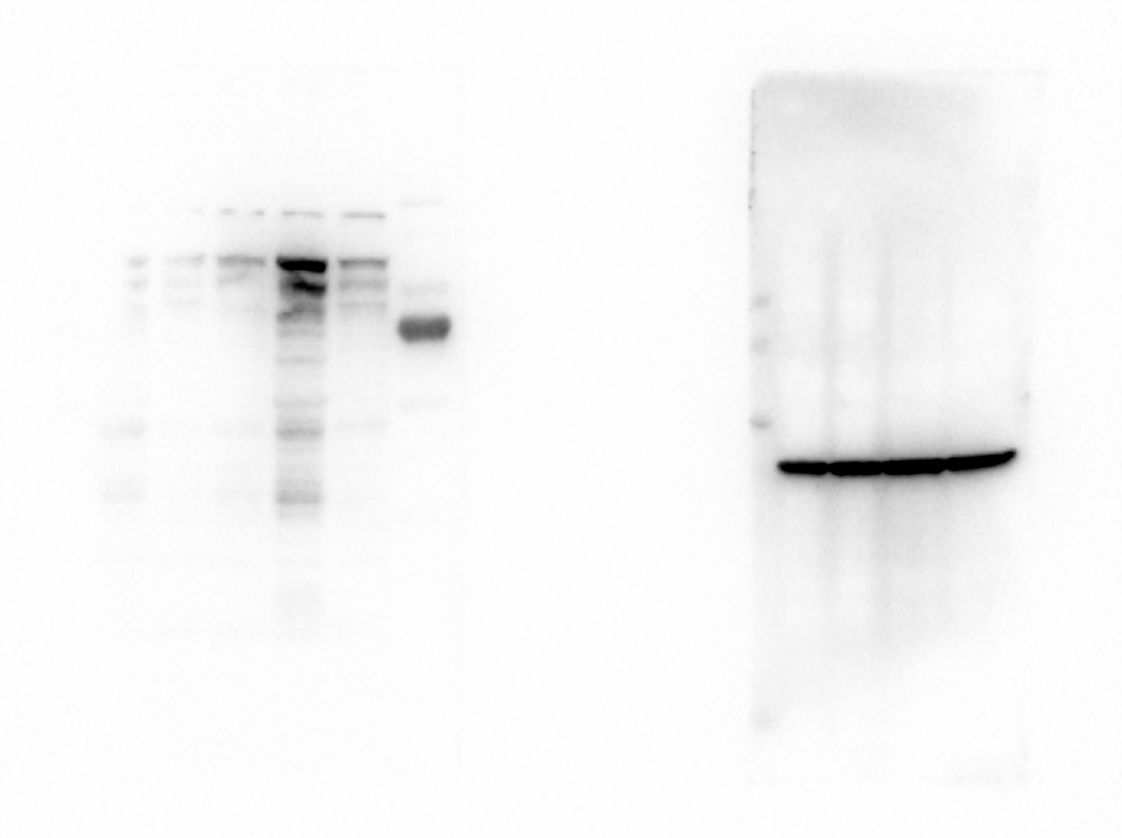

Supplement: Figure 3—figure supplement 2—source data 2. [file elife-84391-fig3-figsupp2-data2.zip › Figure 3-figure supplement 2-source data 2/Figure 3-figure supplement 2B-unmodified blot for anti-AP2B1 anti-actin.tif]

Figure 3-figure supplement 2C

uncropped blots

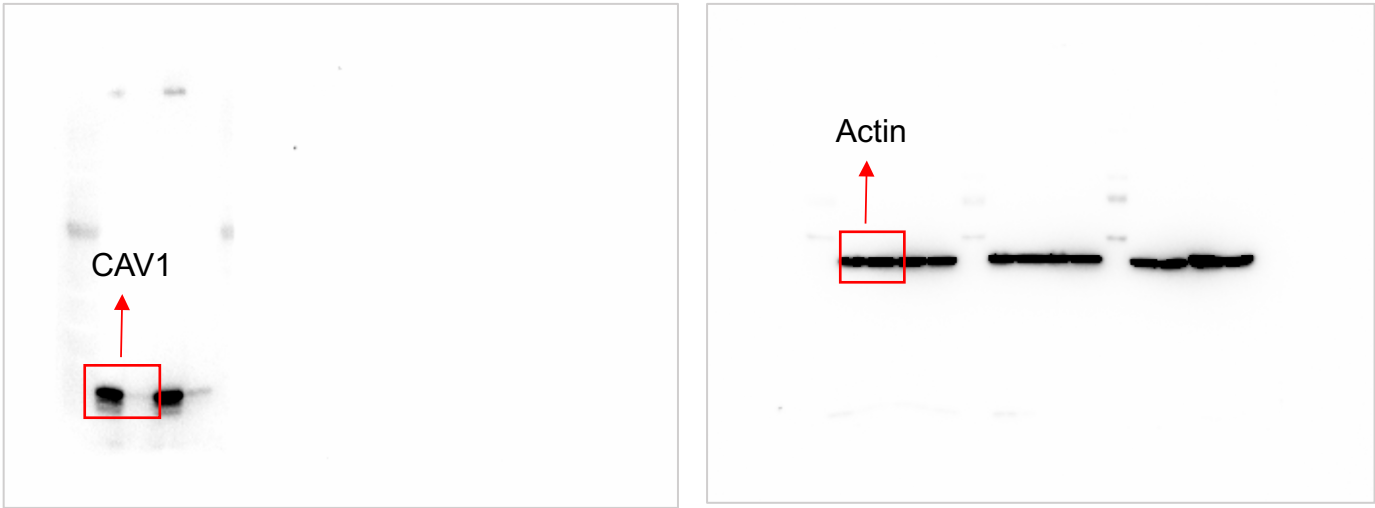

Note: the other lanes are for other experiments.

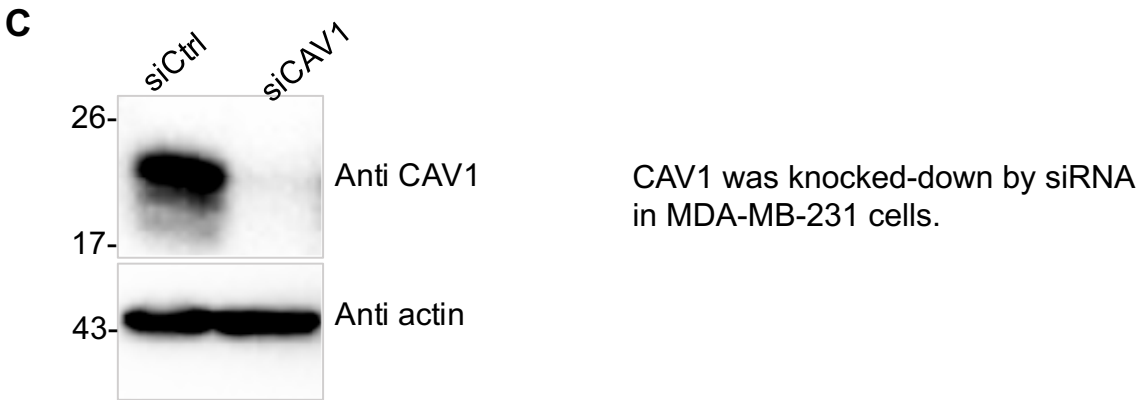

Supplement: Figure 3—figure supplement 2—source data 3. [file elife-84391-fig3-figsupp2-data3.zip › Figure 3-figure supplement 2-source data 3/Figure 3-figure supplement 2-source data 3.pdf]

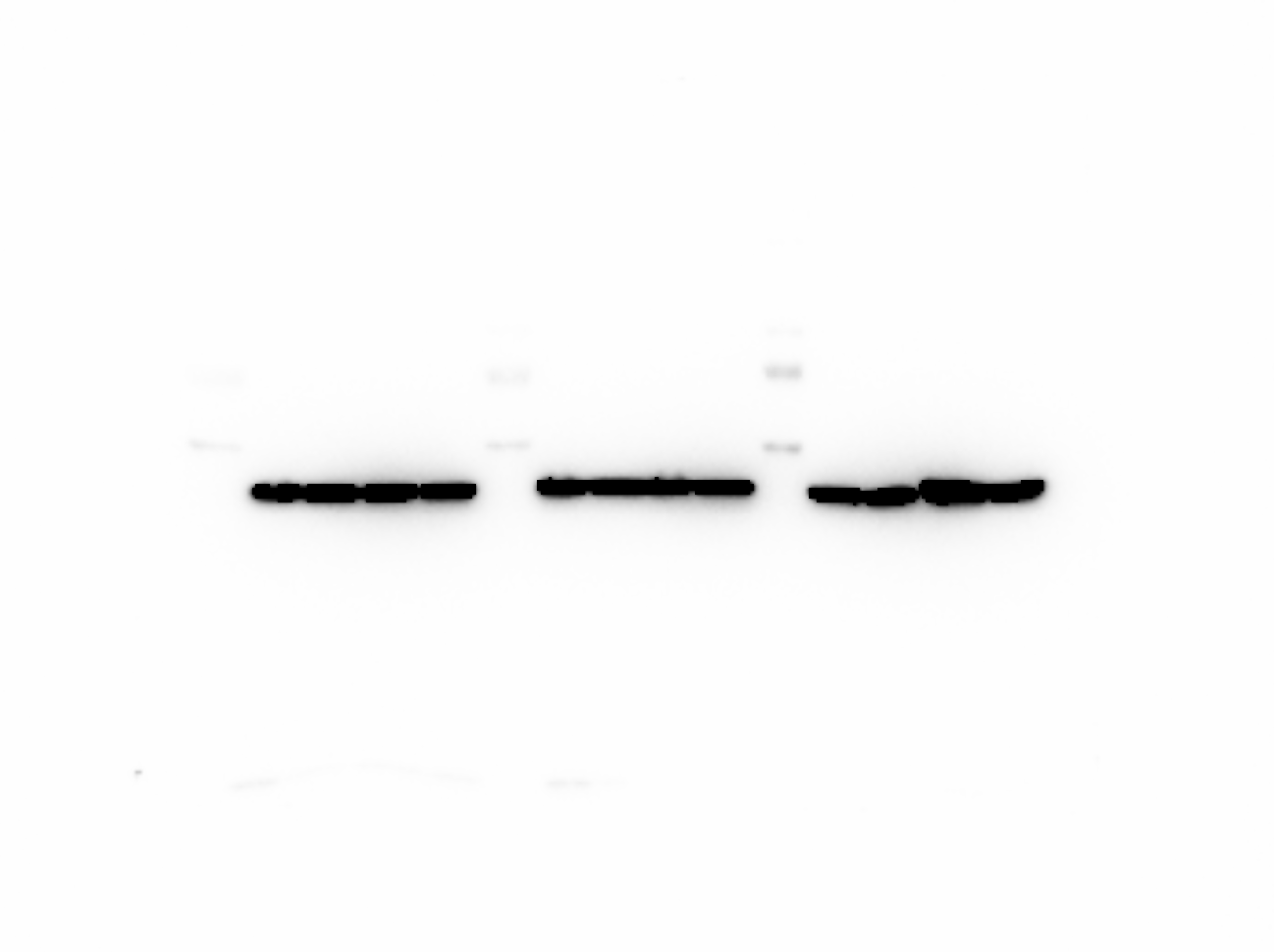

Supplement: Figure 3—figure supplement 2—source data 3. [file elife-84391-fig3-figsupp2-data3.zip › Figure 3-figure supplement 2-source data 3/Figure 3-figure supplement 2C-unmodified blot for anti-actin.tif]

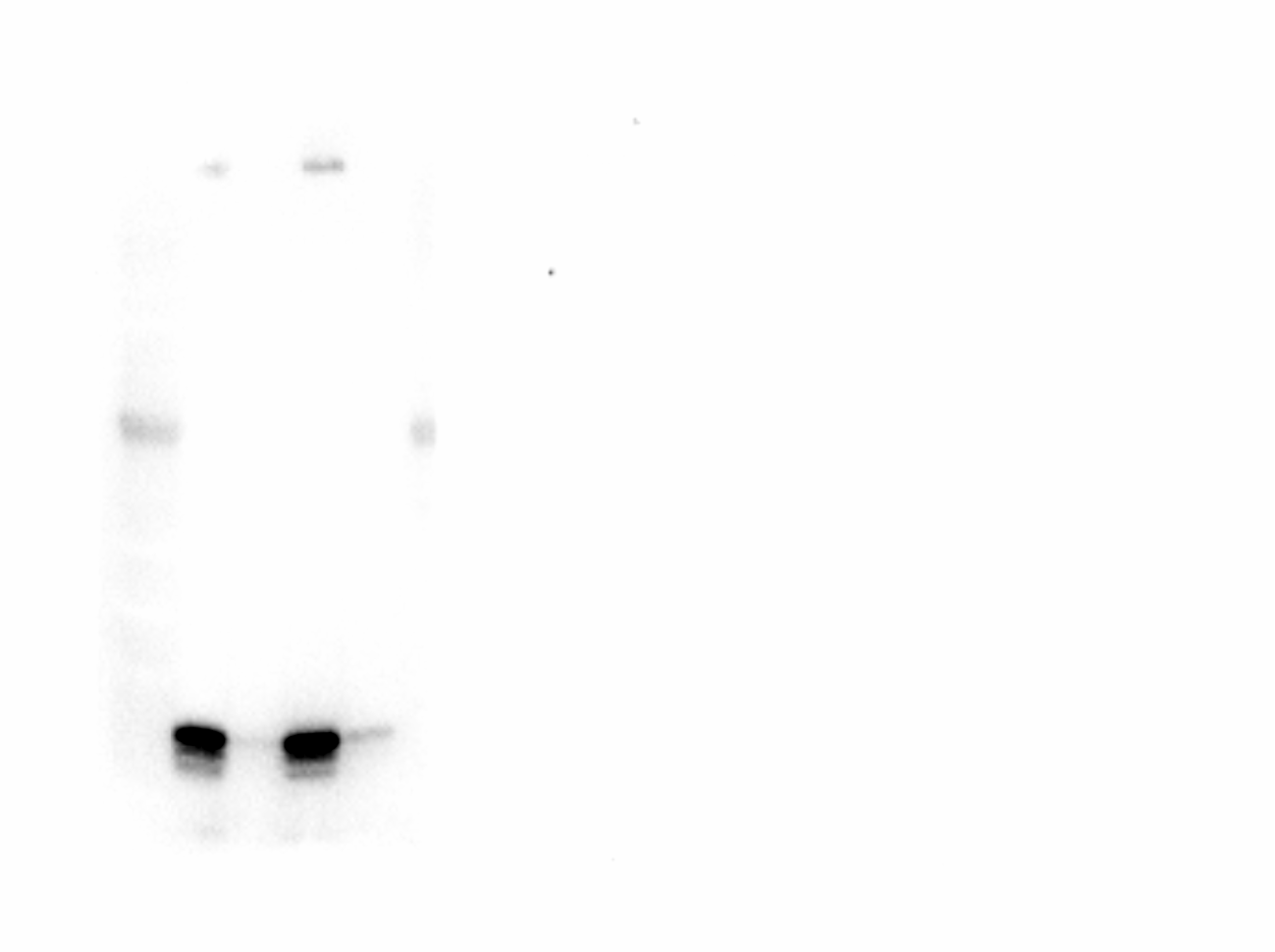

Supplement: Figure 3—figure supplement 2—source data 3. [file elife-84391-fig3-figsupp2-data3.zip › Figure 3-figure supplement 2-source data 3/Figure 3-figure supplement 2C-unmodified blot for anti-CAV1.tif]

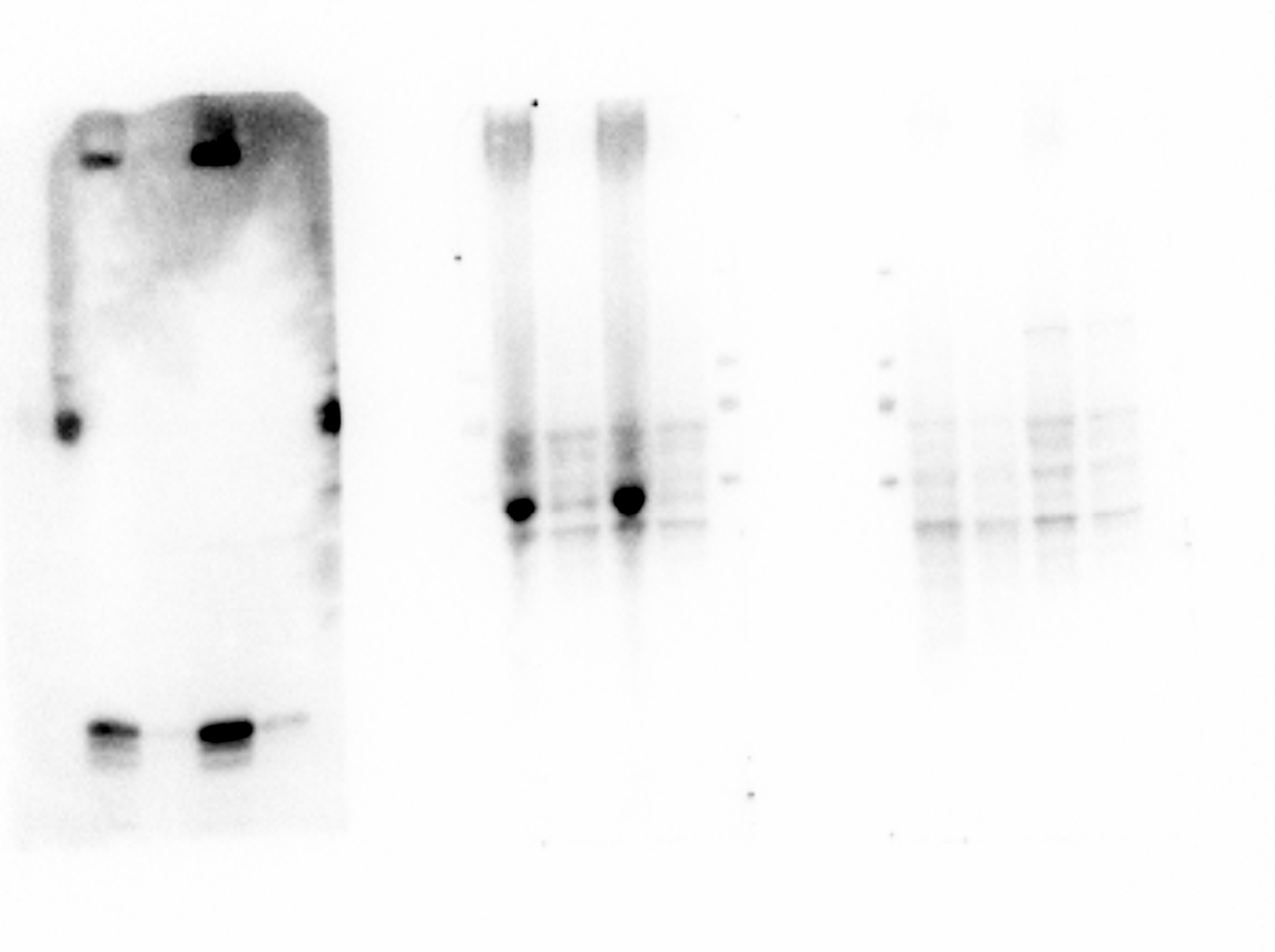

Supplement: Figure 3—figure supplement 2—source data 4. [file elife-84391-fig3-figsupp2-data4.zip › Figure 3-figure supplement 2-source data 4/Figure 3-figure supplement 2D-unmodified blot for anti-FLOT2.tif]

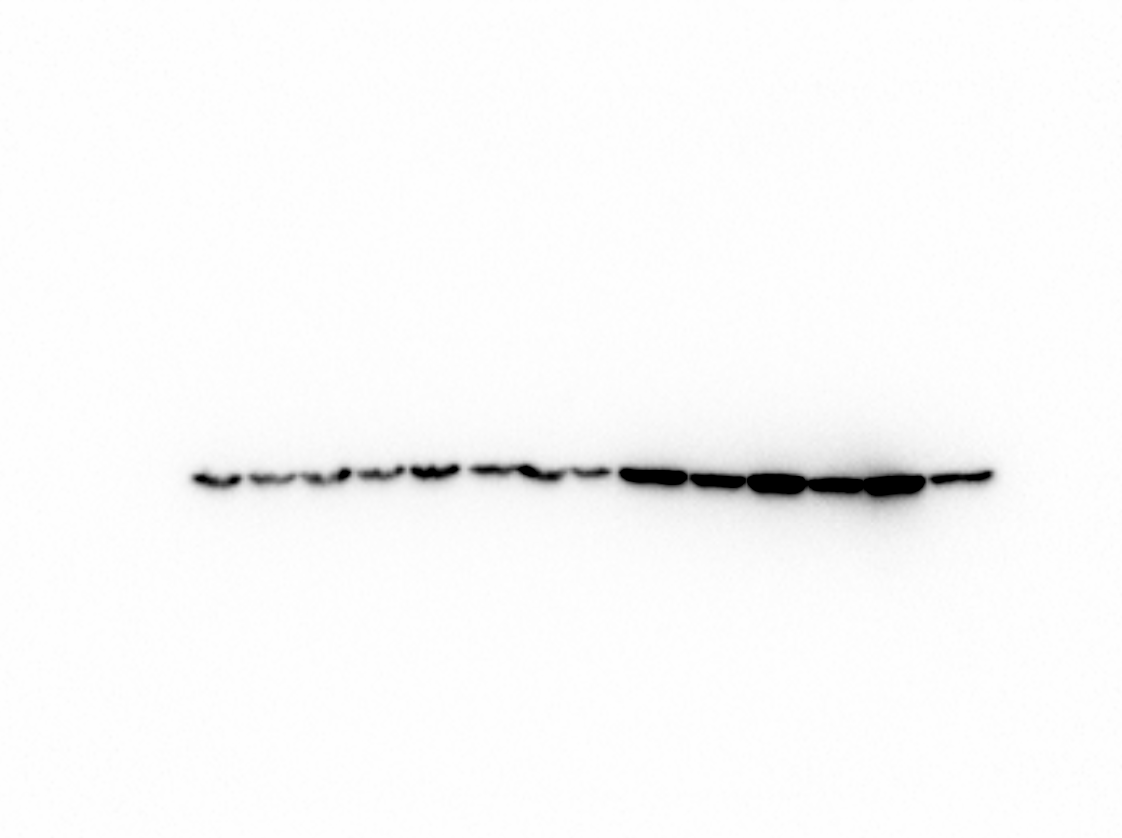

Supplement: Figure 3—figure supplement 2—source data 5. [file elife-84391-fig3-figsupp2-data5.zip › Figure 3-figure supplement 2-source data 5/Figure 3-figure supplement 2E 2H-unmodified blot for anti-actin long exposure.tif]

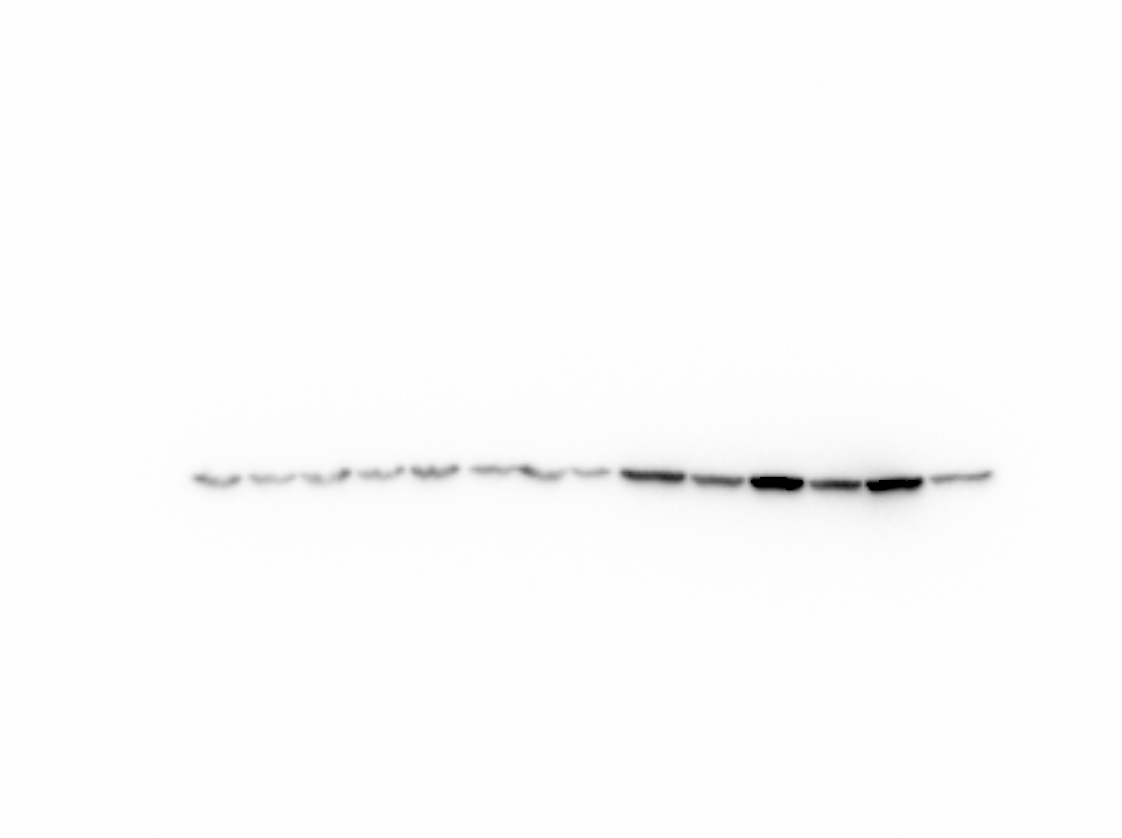

Supplement: Figure 3—figure supplement 2—source data 5. [file elife-84391-fig3-figsupp2-data5.zip › Figure 3-figure supplement 2-source data 5/Figure 3-figure supplement 2E 2H-unmodified blot for anti-actin.tif]

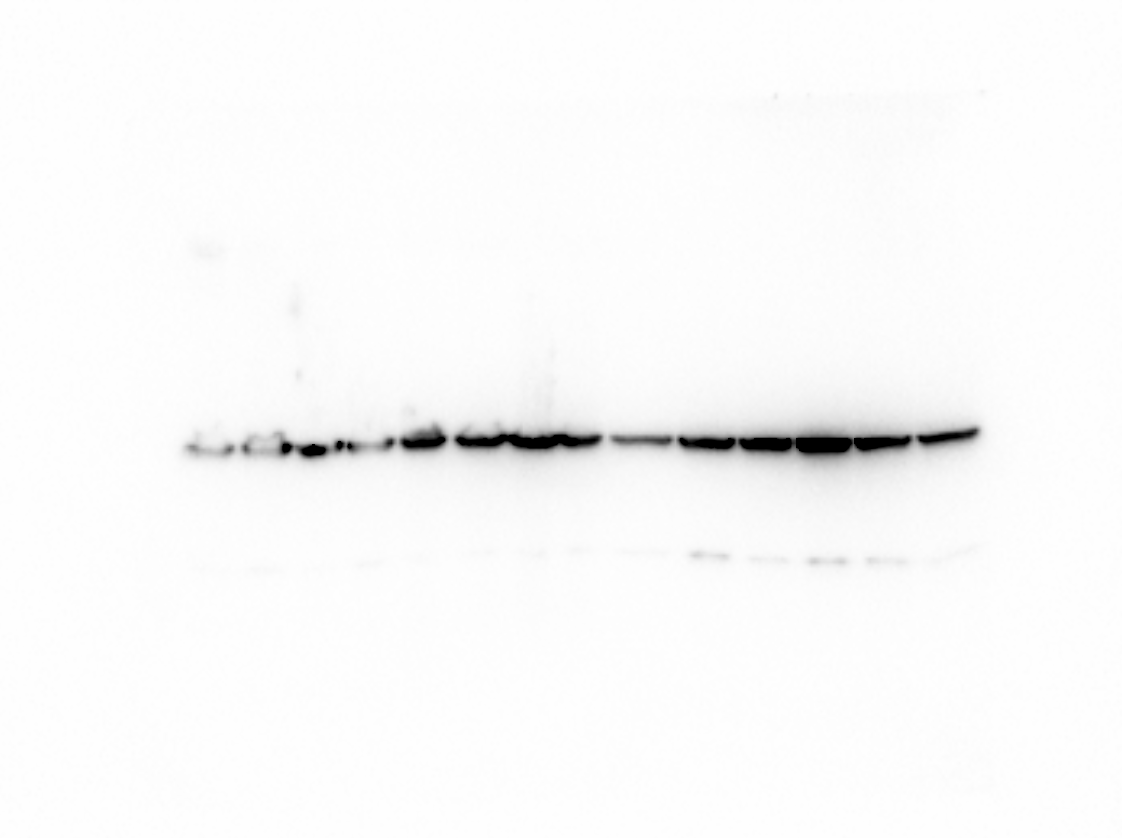

Supplement: Figure 3—figure supplement 2—source data 5. [file elife-84391-fig3-figsupp2-data5.zip › Figure 3-figure supplement 2-source data 5/Figure 3-figure supplement 2E 2H-unmodified blot for anti-tubulin.tif]

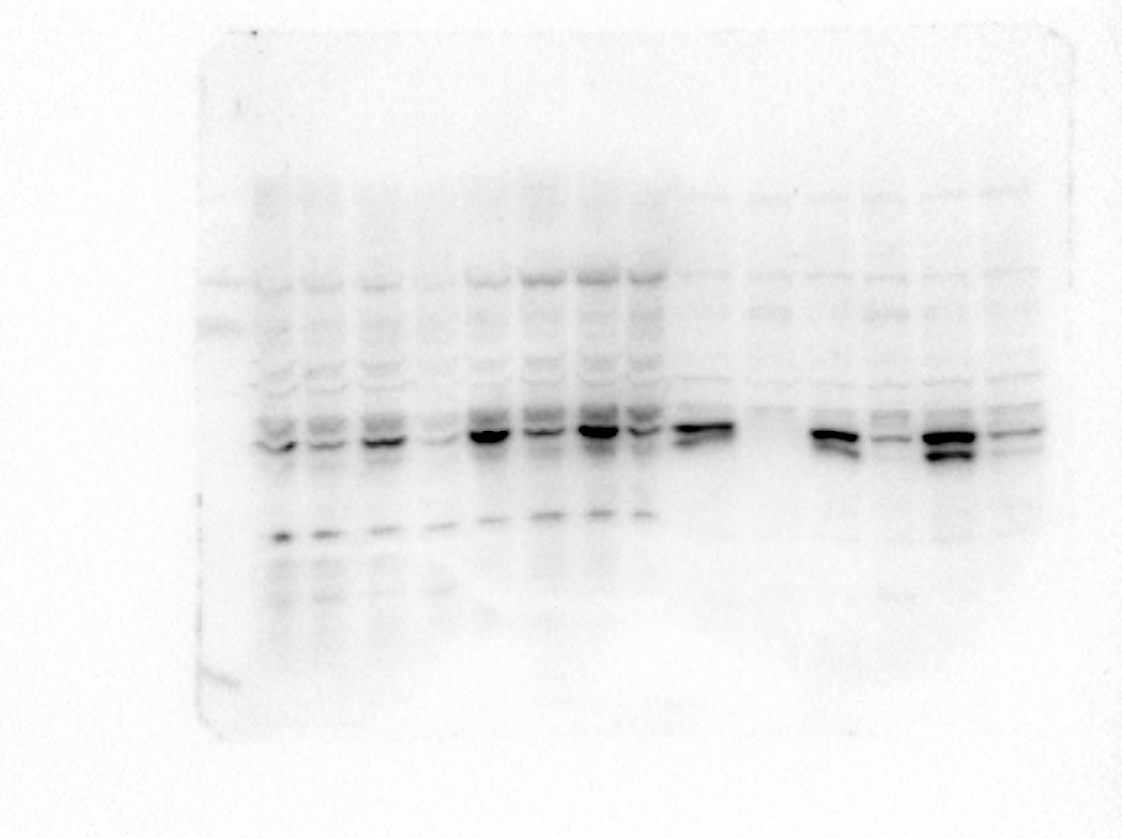

Supplement: Figure 3—figure supplement 2—source data 6. [file elife-84391-fig3-figsupp2-data6.zip › Figure 3-figure supplement 2-source data 6/Figure 3-figure supplement 2F 2I-unmodified blot for anti-Arp2.tif]

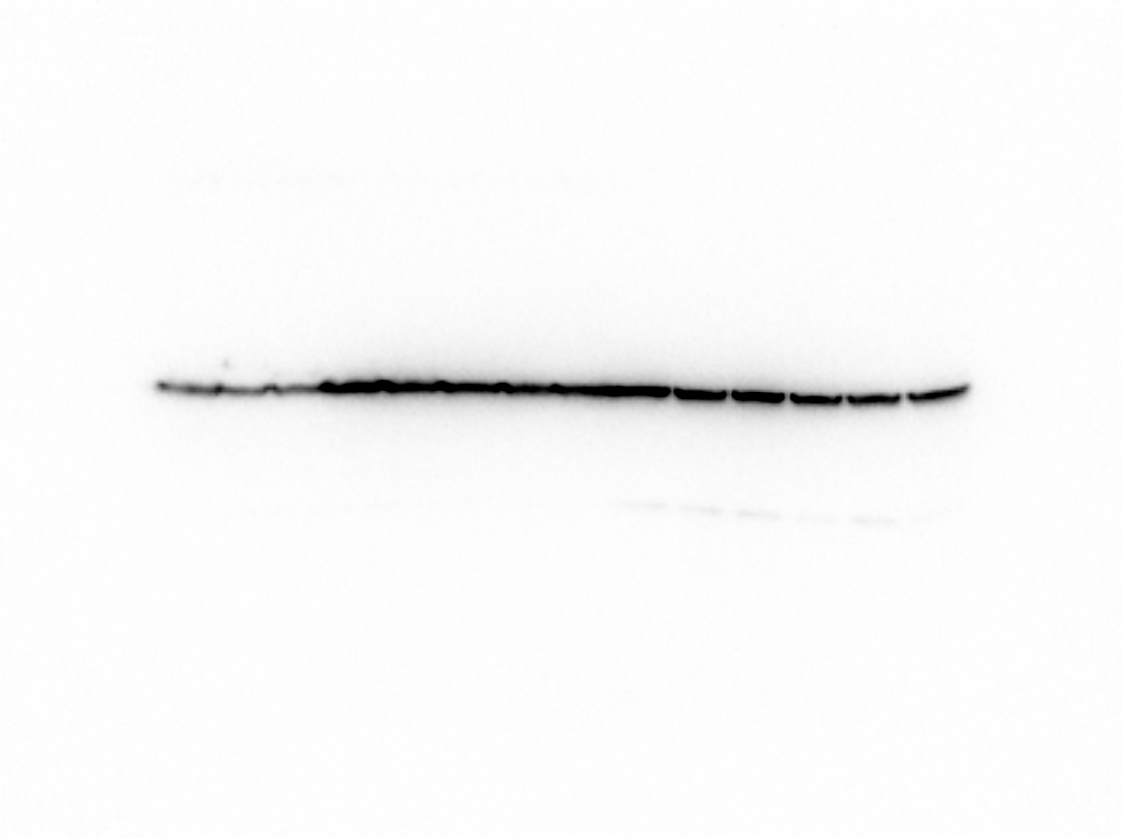

Supplement: Figure 3—figure supplement 2—source data 6. [file elife-84391-fig3-figsupp2-data6.zip › Figure 3-figure supplement 2-source data 6/Figure 3-figure supplement 2F 2I-unmodified blot for anti-tubulin.tif]

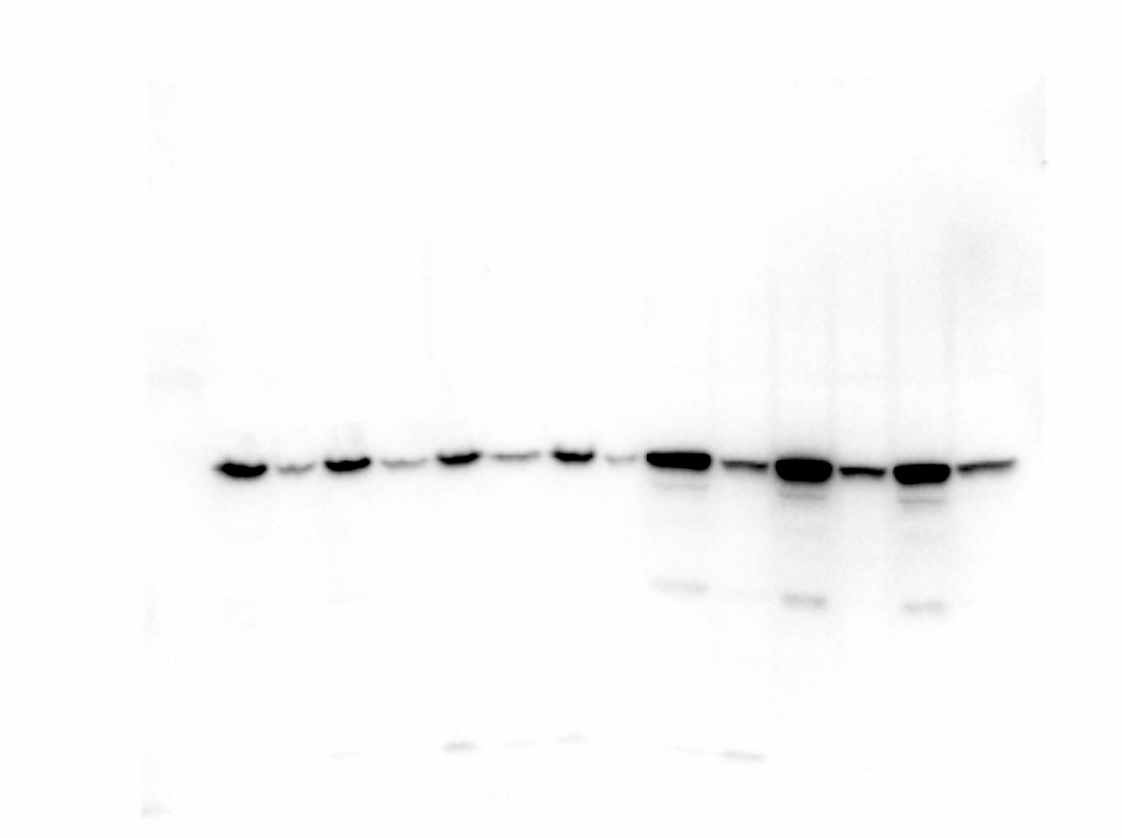

Supplement: Figure 3—figure supplement 2—source data 7. [file elife-84391-fig3-figsupp2-data7.zip › Figure 3-figure supplement 2-source data 7/Figure 3-figure supplement 2G 2J-unmodified blot for anti-Arp3.tif]

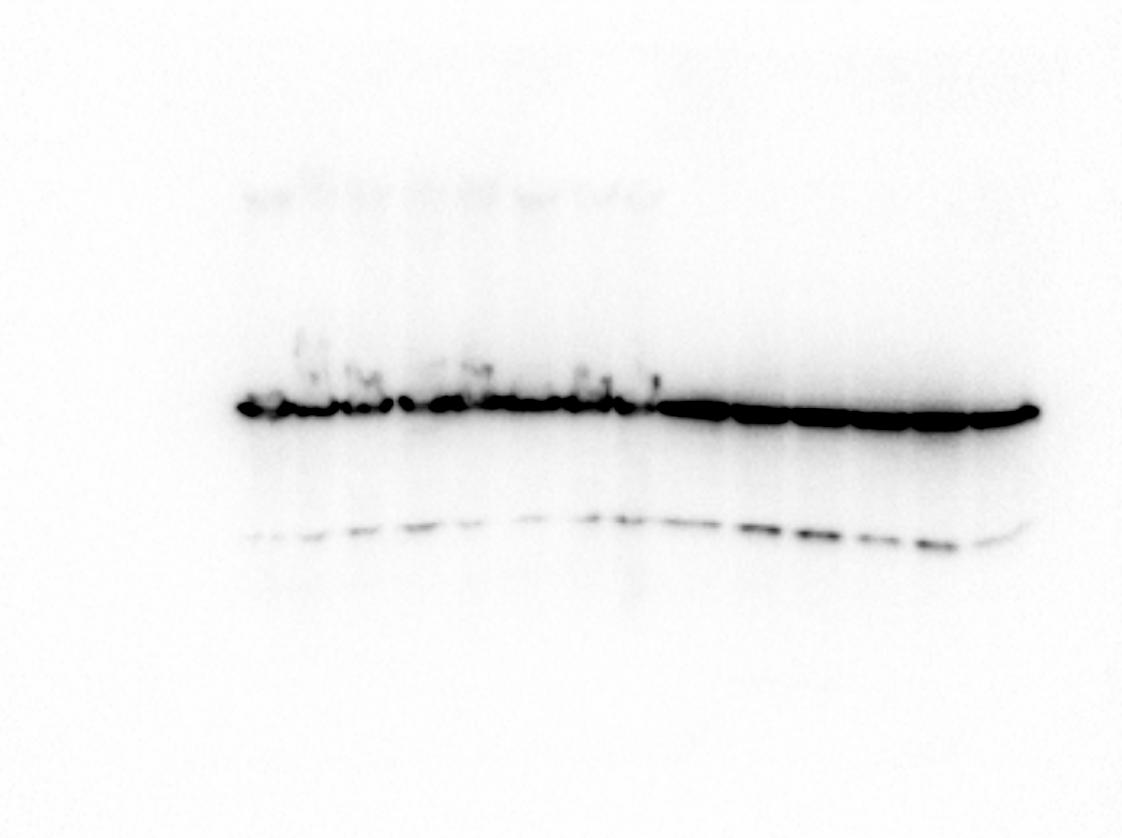

Supplement: Figure 3—figure supplement 2—source data 7. [file elife-84391-fig3-figsupp2-data7.zip › Figure 3-figure supplement 2-source data 7/Figure 3-figure supplement 2G 2J-unmodified blot for anti-tubulin.tif]

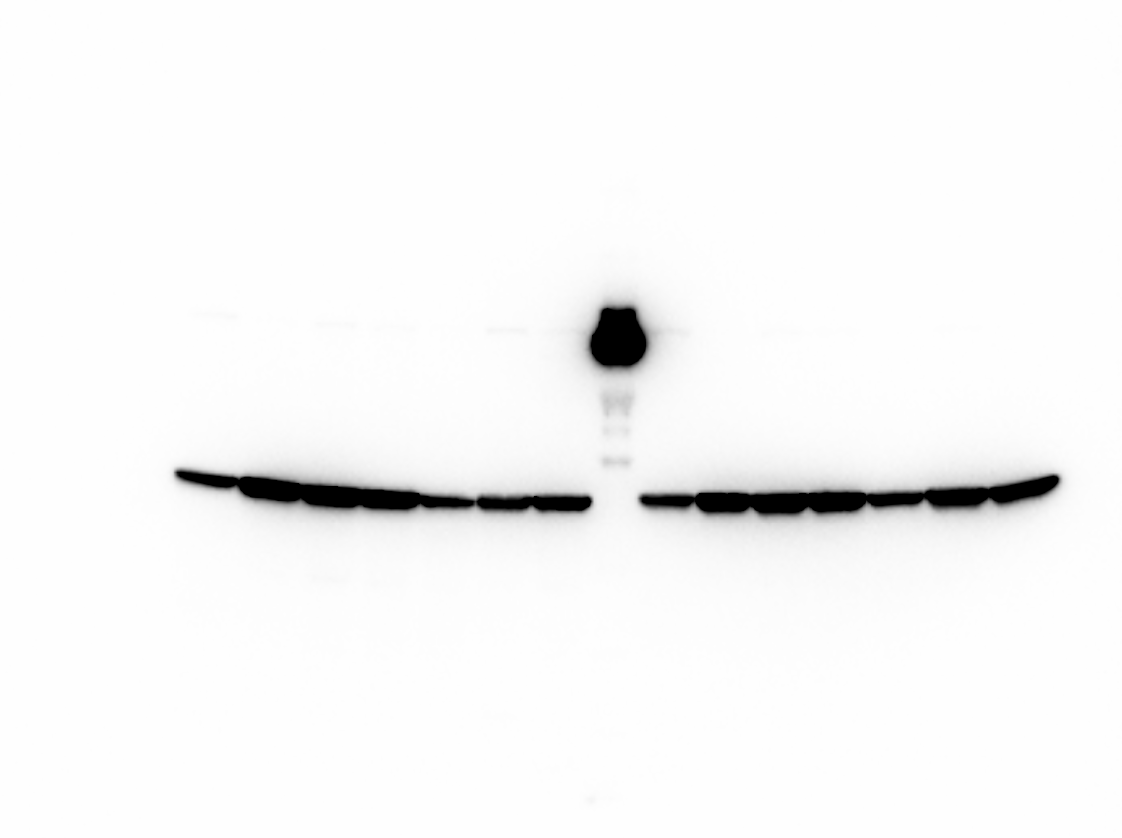

Supplement: Figure 7—figure supplement 1—source data 1. [file elife-84391-fig7-figsupp1-data1.zip › Figure 7-figure supplement 1-source data 1/Figure 7-figure supplement 1A-unmodified blot for anti-actin.tif]

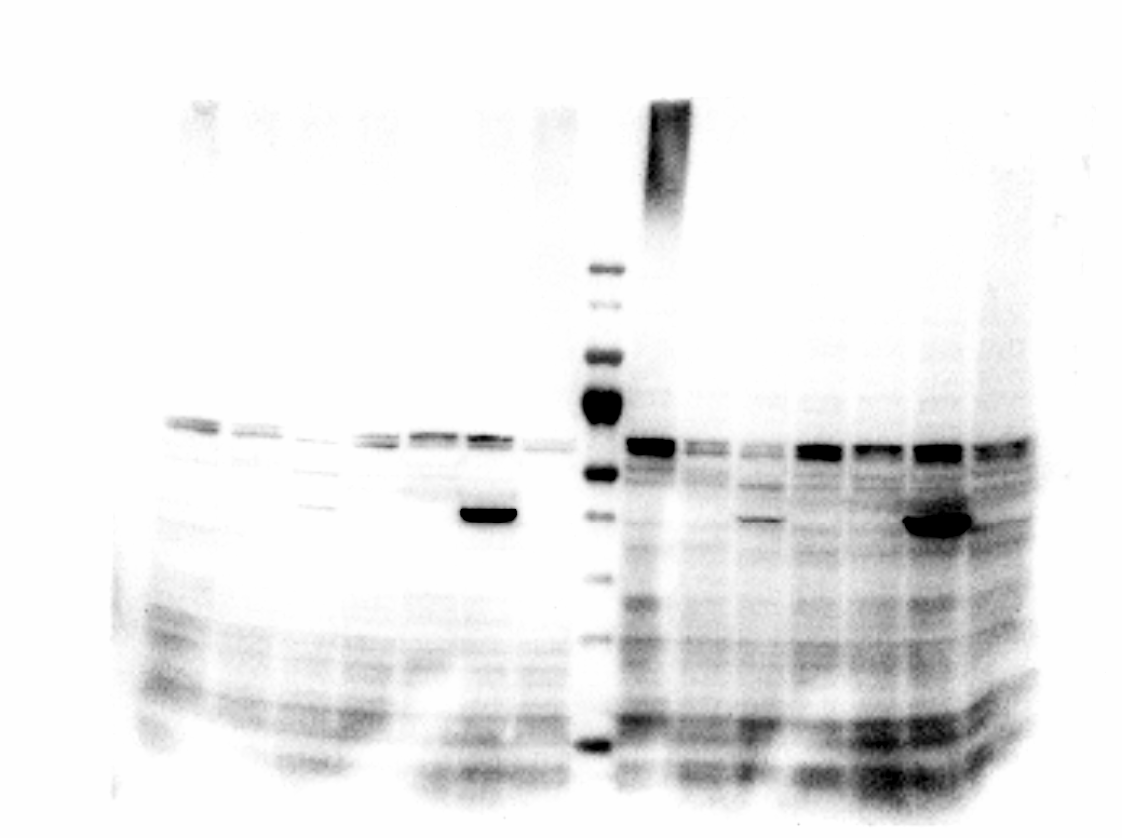

Supplement: Figure 7—figure supplement 1—source data 1. [file elife-84391-fig7-figsupp1-data1.zip › Figure 7-figure supplement 1-source data 1/Figure 7-figure supplement 1A-unmodified blot for anti-syncytin-1.tif]

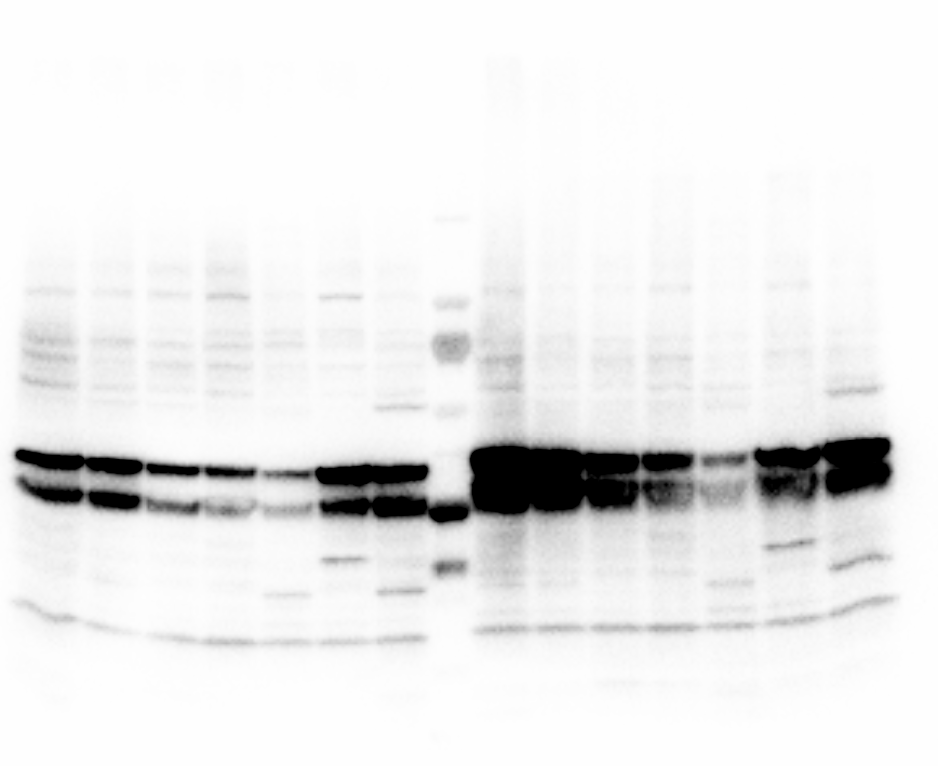

Supplement: Figure 7—figure supplement 1—source data 1. [file elife-84391-fig7-figsupp1-data1.zip › Figure 7-figure supplement 1-source data 1/Figure 7-figure supplement 1A-unmodified blot for anti-syncytin-2.tif]

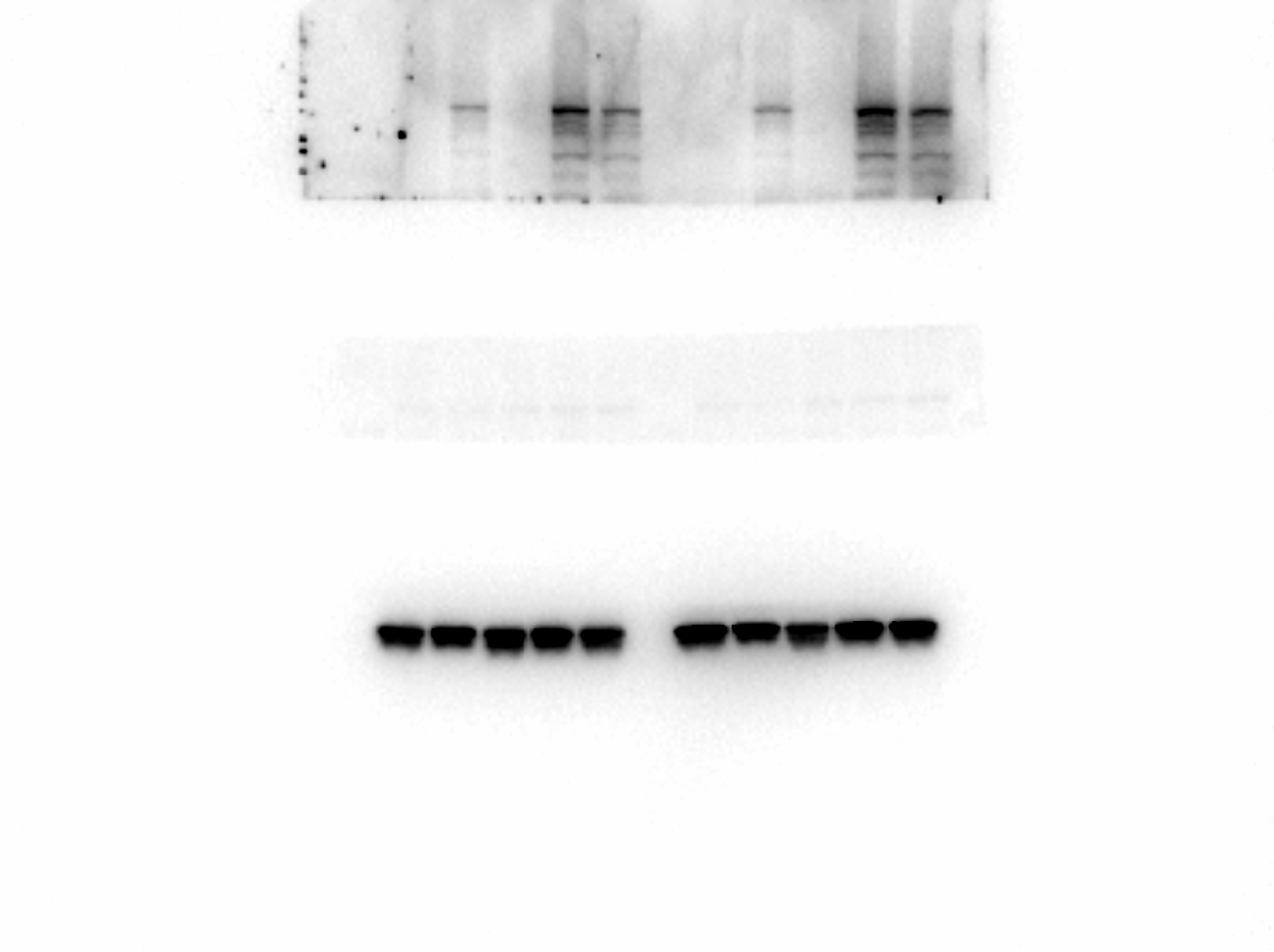

Supplement: Figure 7—figure supplement 1—source data 2. [file elife-84391-fig7-figsupp1-data2.zip › Figure 7-figure supplement 1-source data 2/Figure 7-figure supplement 1B-unmodified blot for anti-Cas9 anti-actin.tif]

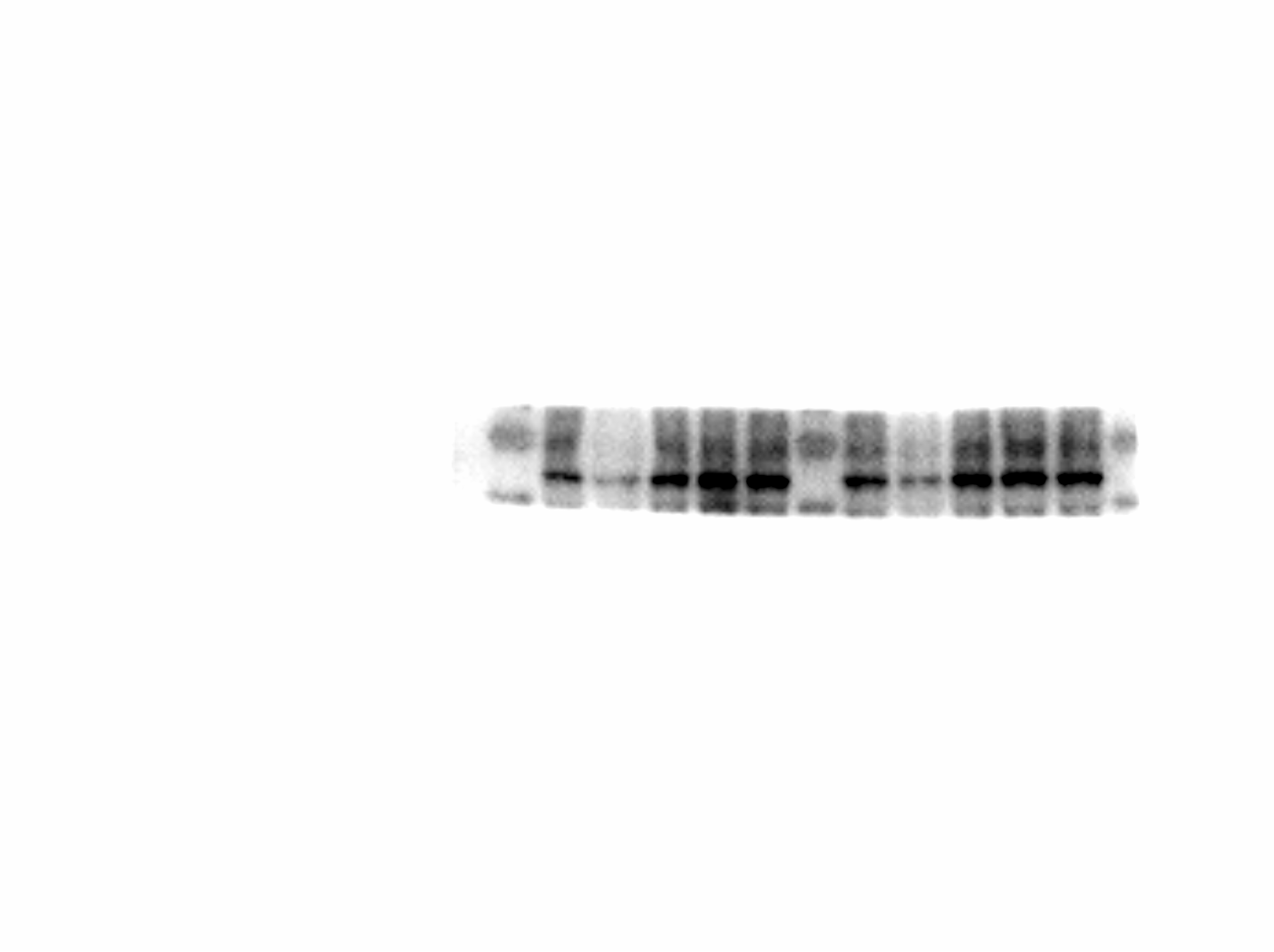

Supplement: Figure 7—figure supplement 1—source data 2. [file elife-84391-fig7-figsupp1-data2.zip › Figure 7-figure supplement 1-source data 2/Figure 7-figure supplement 1B-unmodified blot for anti-syncytin-1.tif]

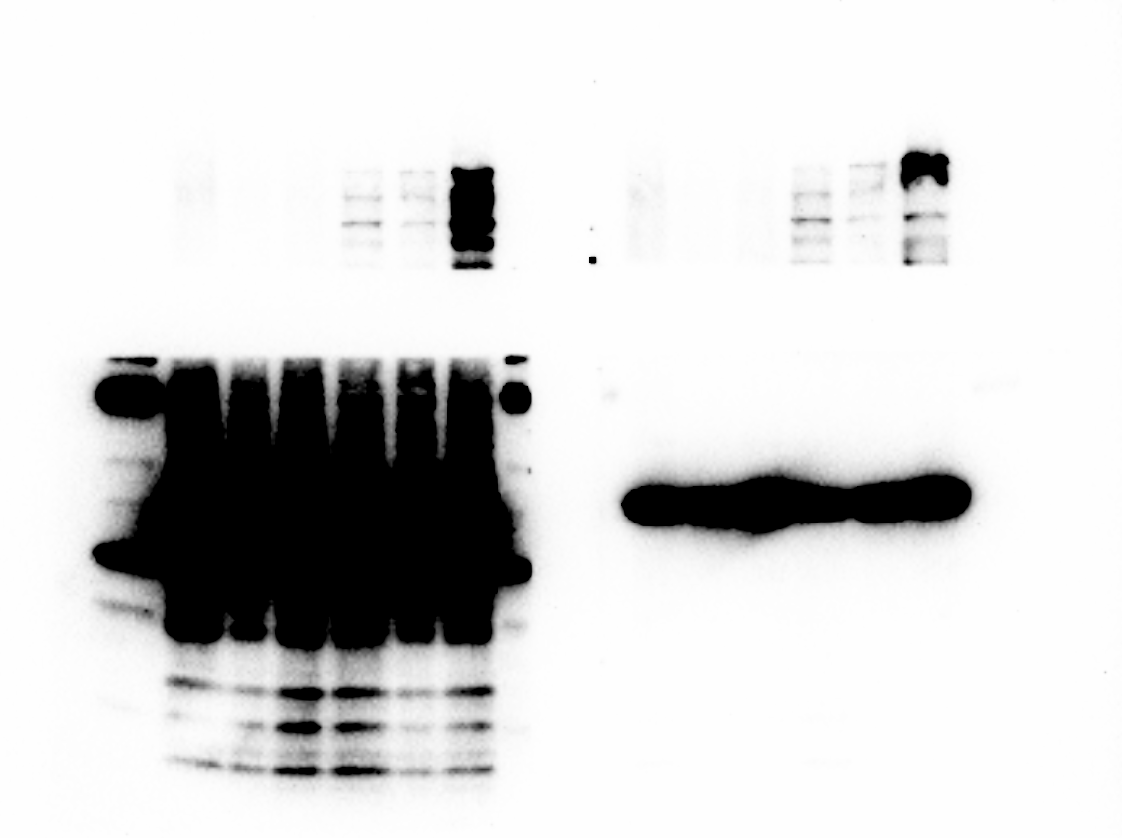

Supplement: Figure 7—figure supplement 1—source data 3. [file elife-84391-fig7-figsupp1-data3.zip › Figure 7-figure supplement 1-source data 3/Figure 7-figure supplement 1D-unmodified blot for anti-Cas9 anti-syncytin2 anti-actin long exposure.tif]

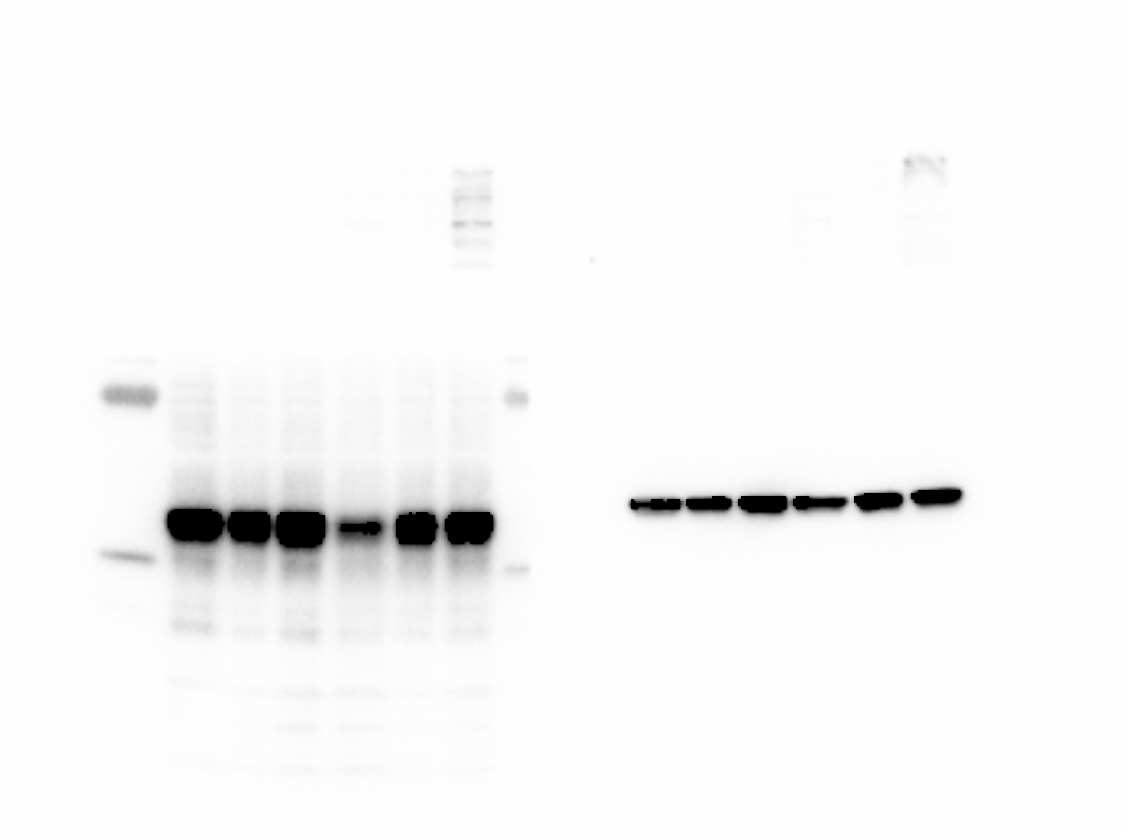

Supplement: Figure 7—figure supplement 1—source data 3. [file elife-84391-fig7-figsupp1-data3.zip › Figure 7-figure supplement 1-source data 3/Figure 7-figure supplement 1D-unmodified blot for anti-Cas9 anti-syncytin2 anti-actin.tif]

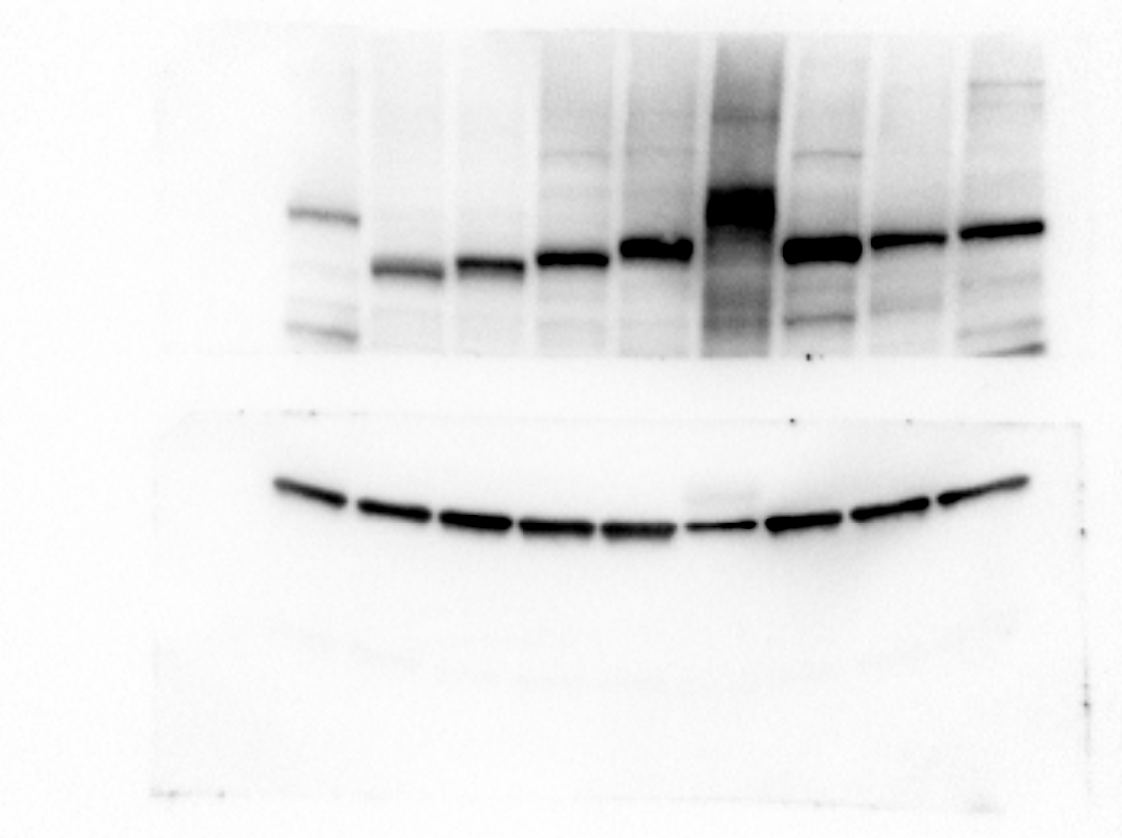

Supplement: Figure 7—figure supplement 1—source data 4. [file elife-84391-fig7-figsupp1-data4.zip › Figure 7-figure supplement 1-source data 4/Figure 7-figure supplement 1F-unmodified blot for anti-GFP.tif]

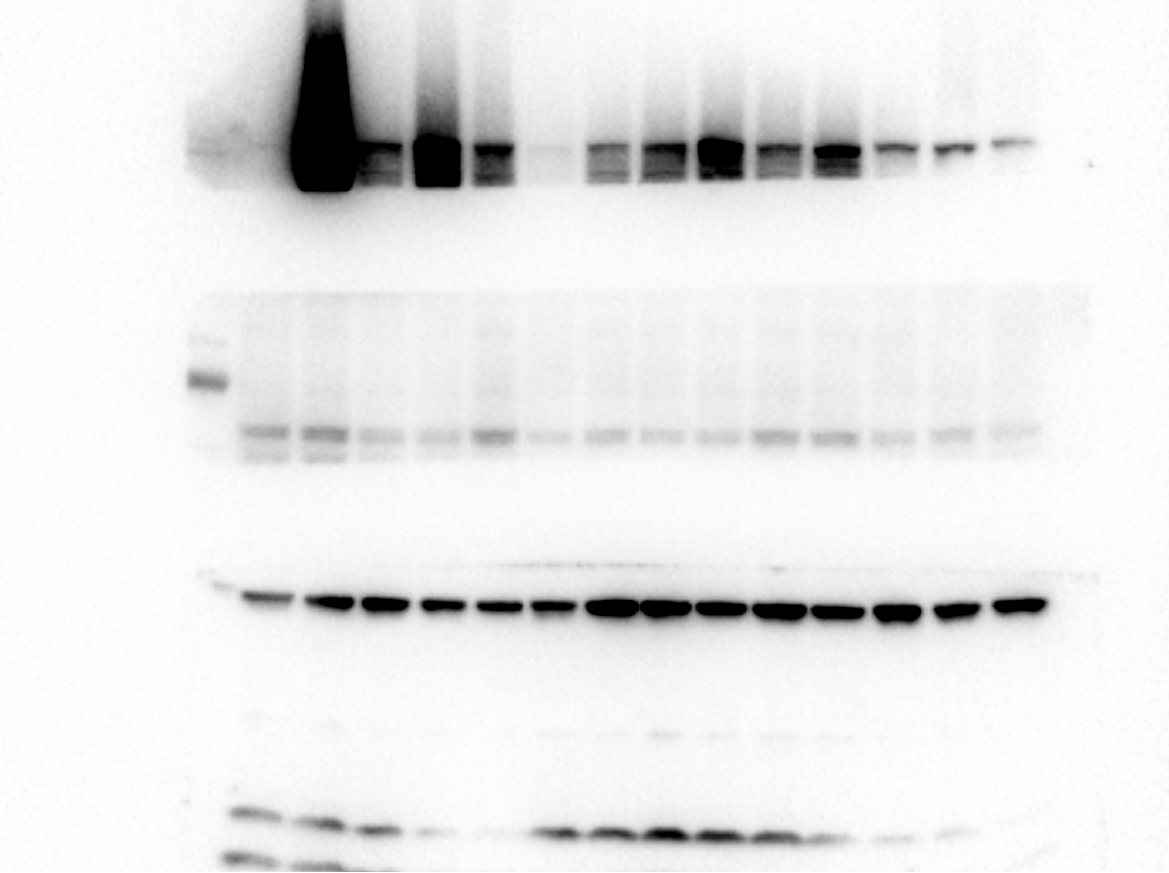

Supplement: Figure 7—figure supplement 1—source data 5. [file elife-84391-fig7-figsupp1-data5.zip › Figure 7-figure supplement 1-source data 5/Figure 7-figure supplement 1G-unmodified blot for anti-syncytin-1 anti-actin short exposure.tif]

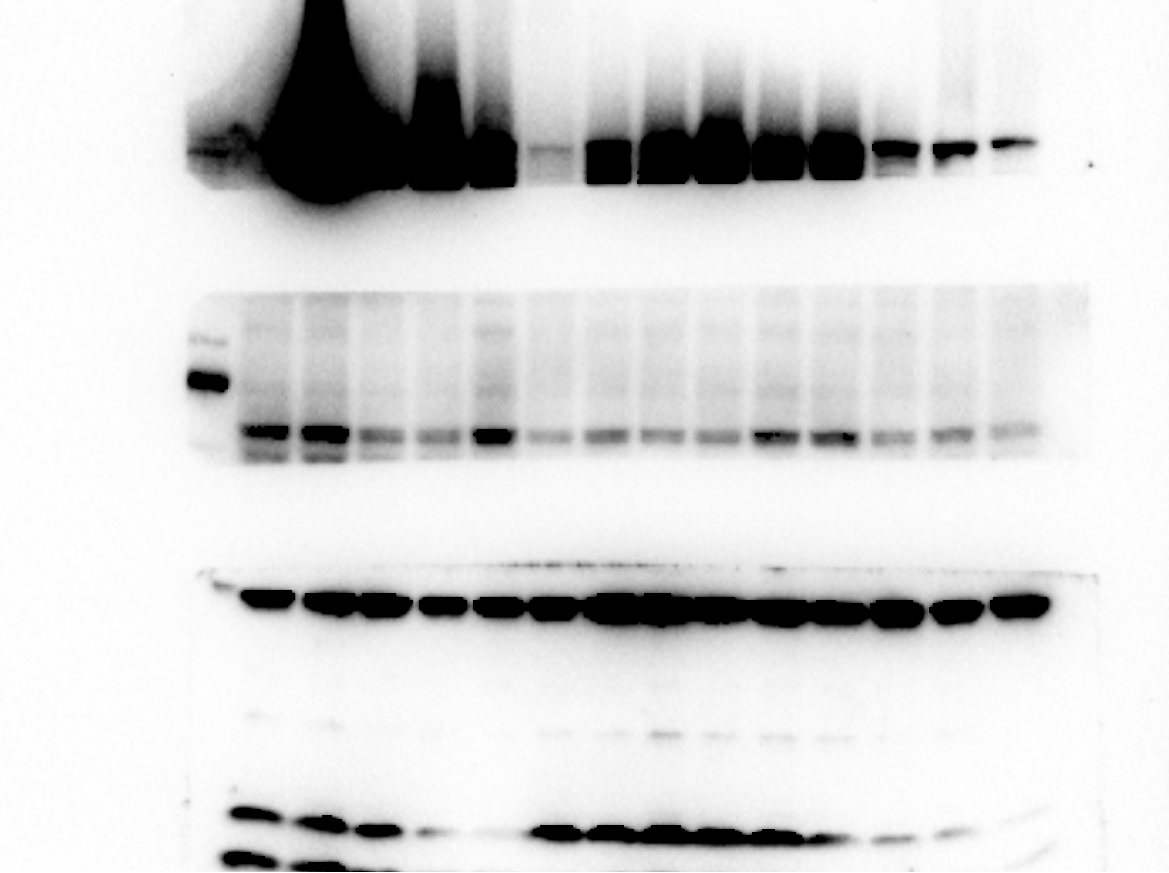

Supplement: Figure 7—figure supplement 1—source data 5. [file elife-84391-fig7-figsupp1-data5.zip › Figure 7-figure supplement 1-source data 5/Figure 7-figure supplement 1G-unmodified blot for anti-syncytin-1 anti-actin.tif]

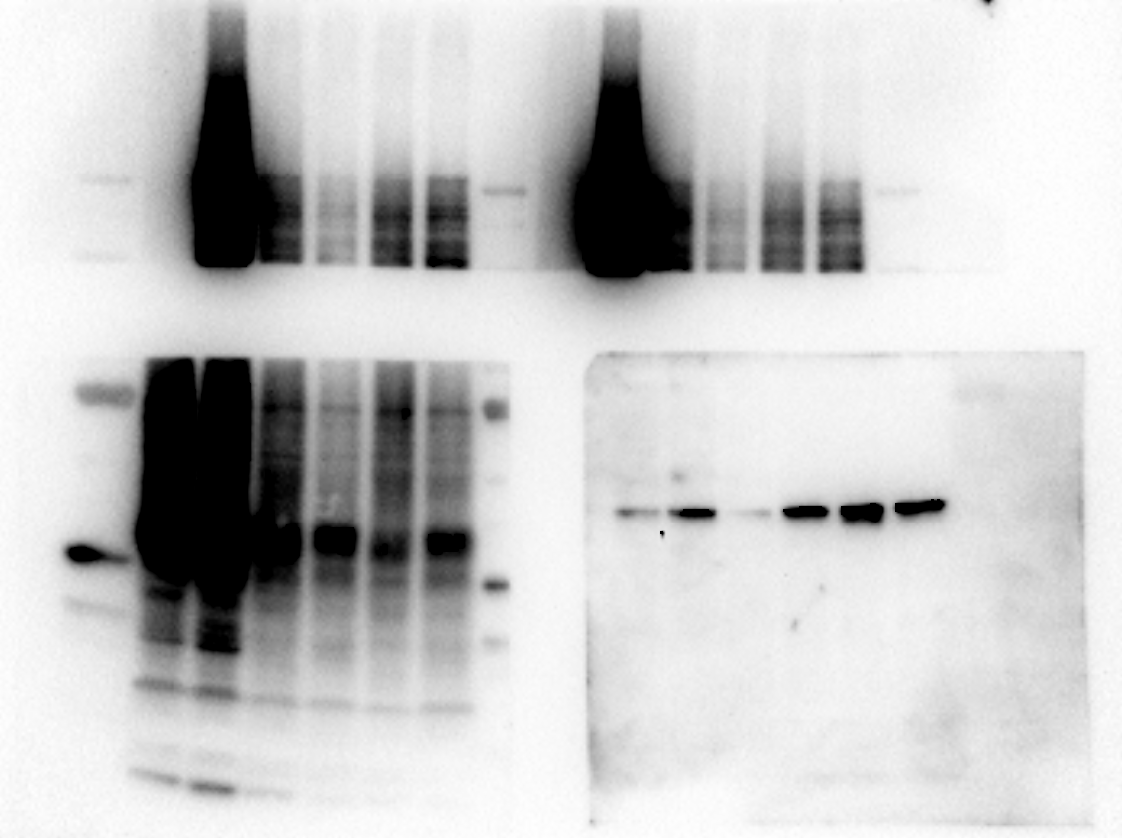

Supplement: Figure 7—figure supplement 1—source data 6. [file elife-84391-fig7-figsupp1-data6.zip › Figure 7-figure supplement 1-source data 6/Figure 7-figure supplement 1H-unmodified blot for anti-syncytin-2 anti-actin long exposure.tif]

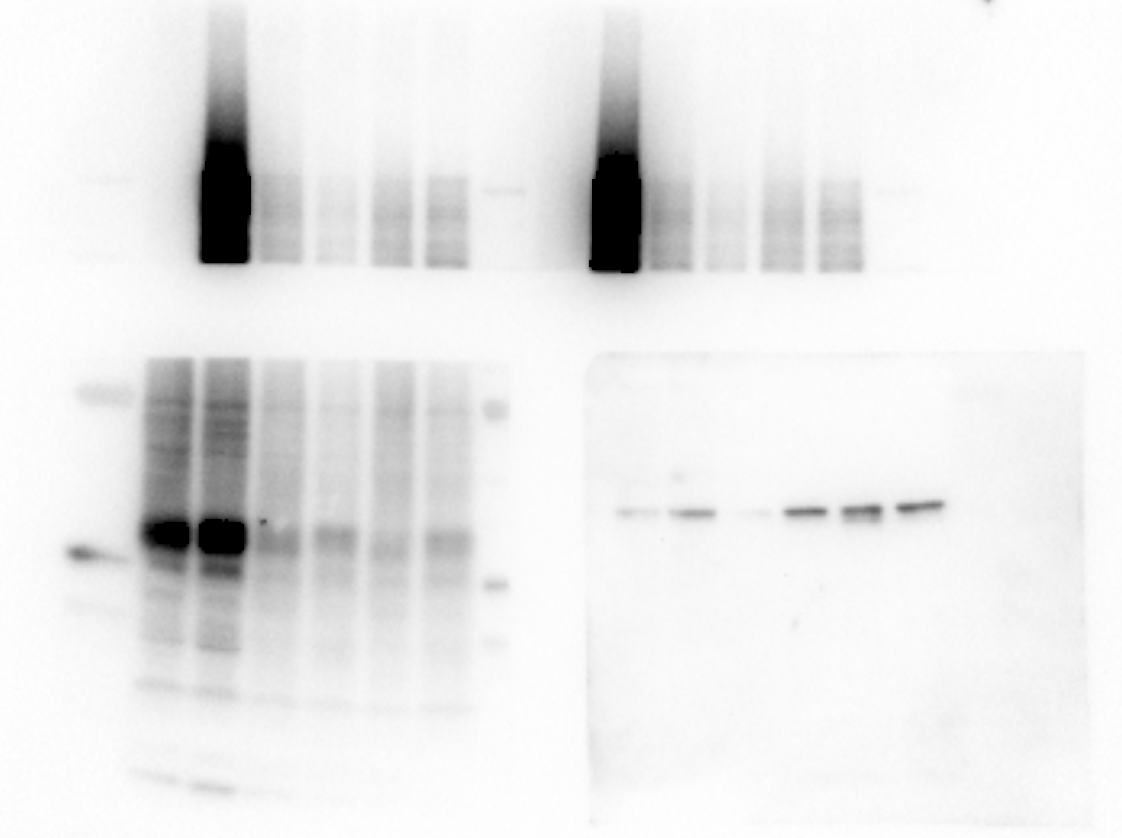

Supplement: Figure 7—figure supplement 1—source data 6. [file elife-84391-fig7-figsupp1-data6.zip › Figure 7-figure supplement 1-source data 6/Figure 7-figure supplement 1H-unmodified blot for anti-syncytin-2 anti-actin.tif]

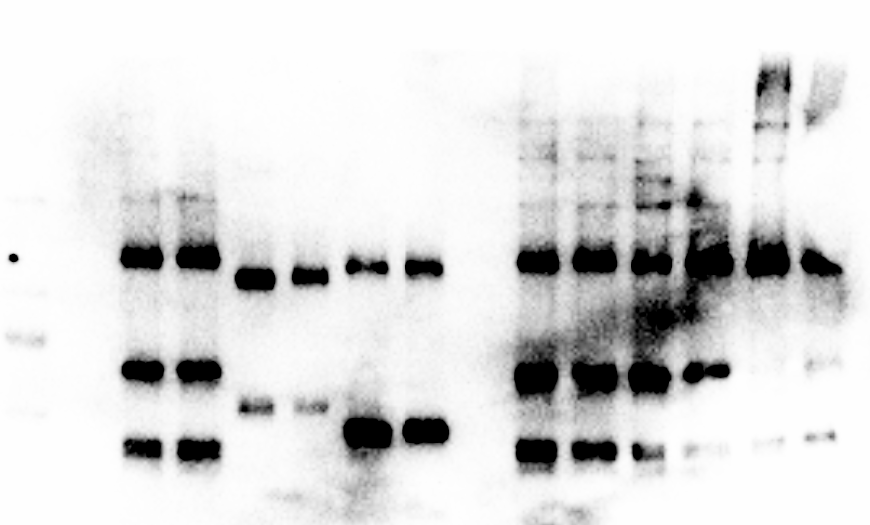

Supplement: Figure 8—figure supplement 1—source data 1. [file elife-84391-fig8-figsupp1-data1.zip › Figure 8-figure supplement 1-source data 1/Figure 8-figure supplement 1D-unmodified blot for anti-GFP.tif]

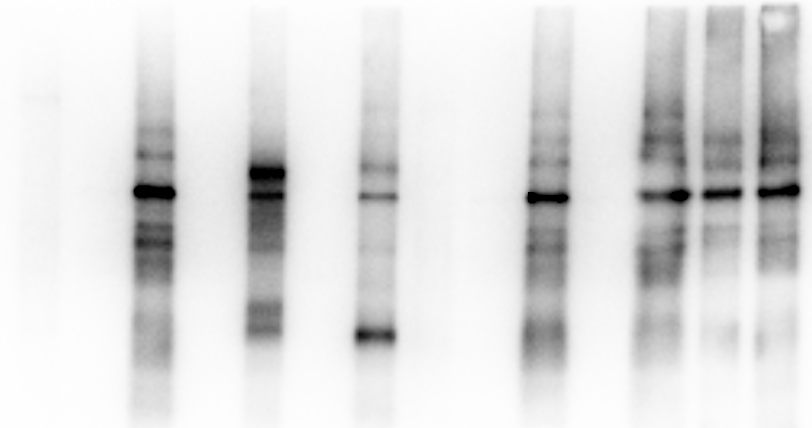

Supplement: Figure 8—figure supplement 1—source data 1. [file elife-84391-fig8-figsupp1-data1.zip › Figure 8-figure supplement 1-source data 1/Figure 8-figure supplement 1D-unmodified blot for Streptavidin-HRP.tif]

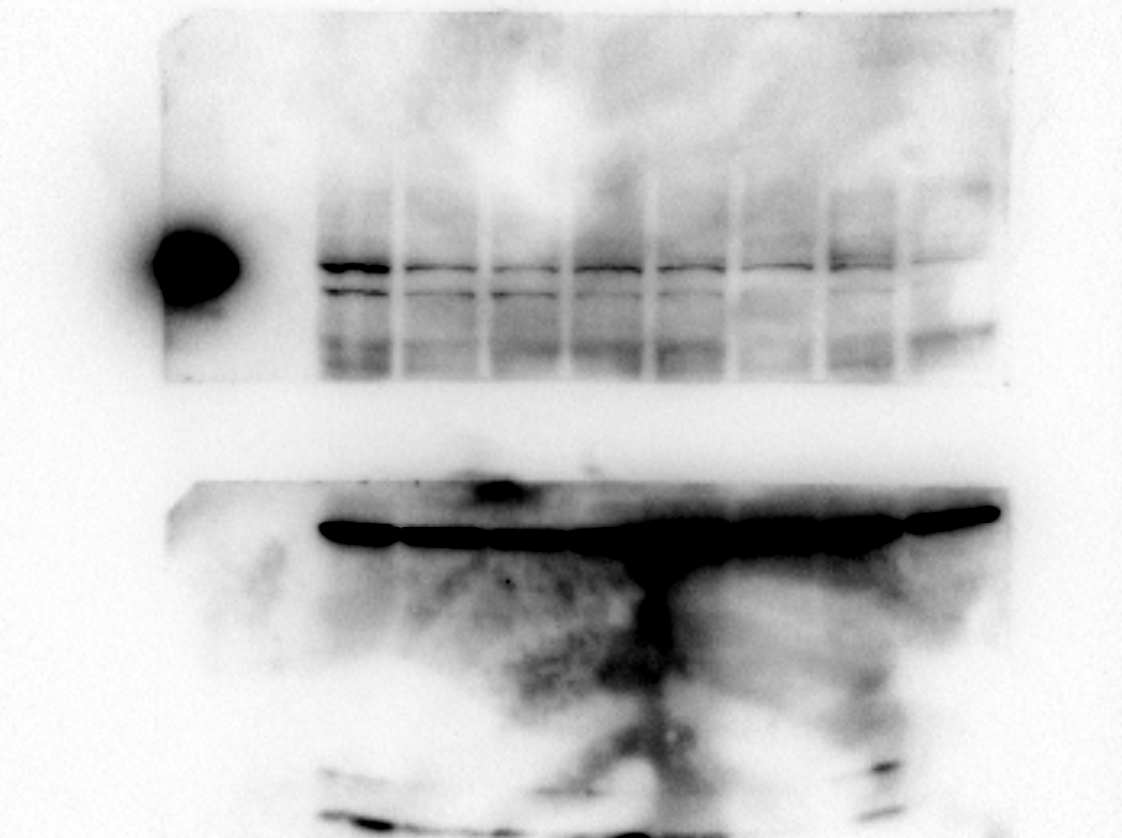

Supplement: Figure 9—source data 1. [file elife-84391-fig9-data1.zip › Figure 9-source data 1/Figure 9B-unmodified blot for anti-MFSD2A anti-actin.tif]

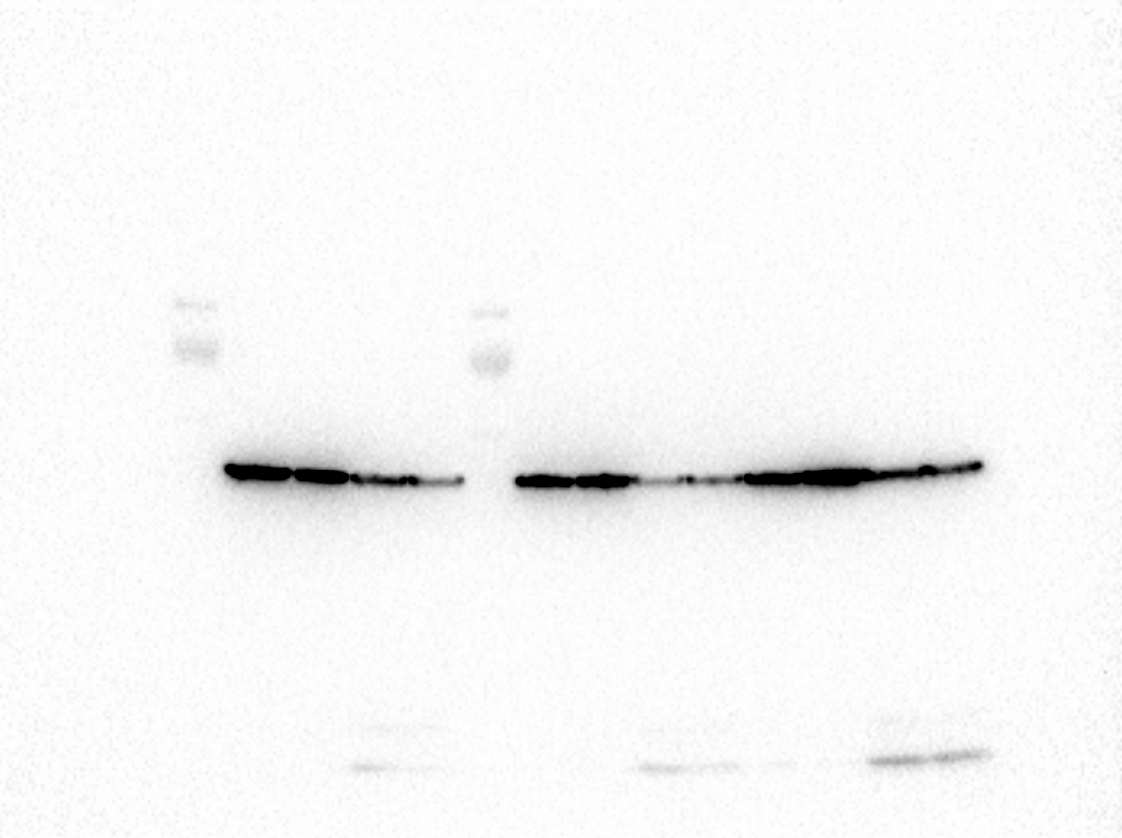

Supplement: Figure 9—source data 2. [file elife-84391-fig9-data2.zip › Figure 9-source data 2/Figure 9D-unmodified blot for anti-actin.tif]

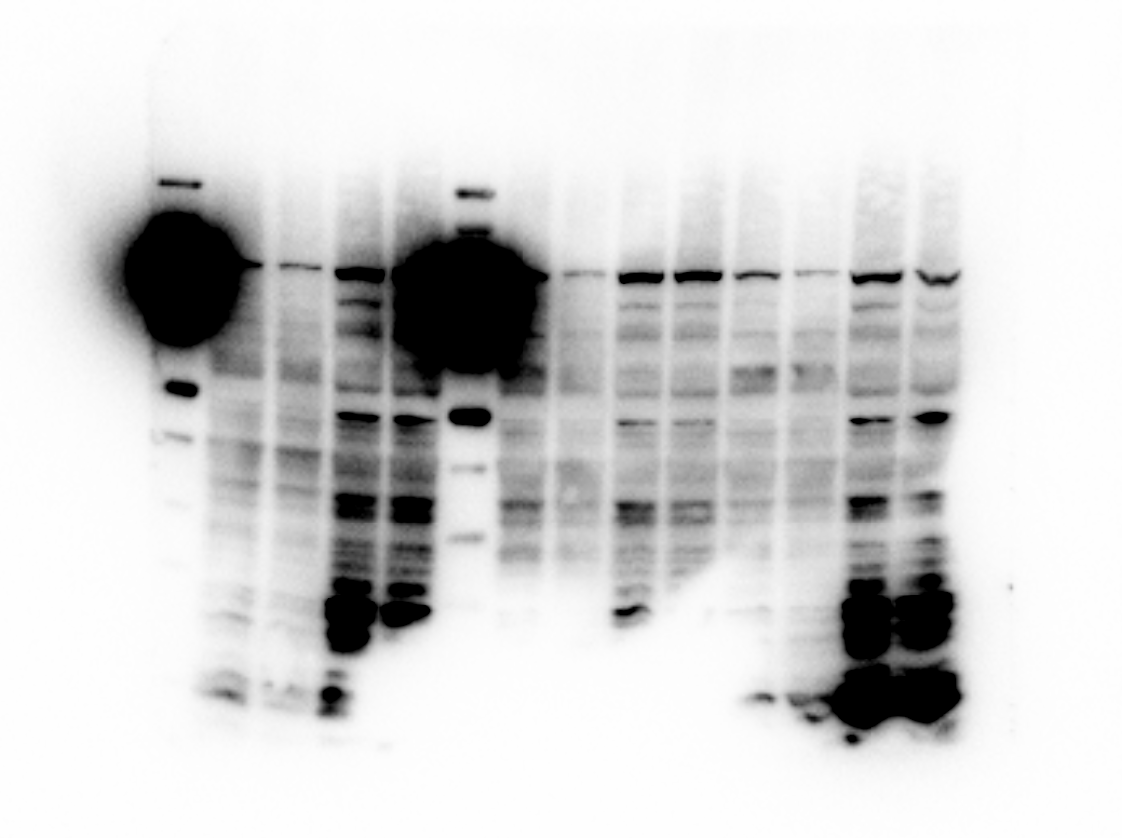

Supplement: Figure 9—source data 2. [file elife-84391-fig9-data2.zip › Figure 9-source data 2/Figure 9D-unmodified blot for anti-MFSD2A.tif]
