# Supplementary material for: Syncytin-mediated open-ended membrane tubular connections facilitate the intercellular transfer of cargos including Cas9 protein
Source: eLife. 2023 Mar 10;12:e84391. doi: 10.7554/eLife.84391 (PMC10112890; doi:10.7554/eLife.84391)
Supplement: Figure 1—figure supplement 1—source data 2. [file elife-84391-fig1-figsupp1-data2.zip › Figure 1-figure supplement 1-source data 2/Figure 1-figure supplement 1-source data 2.pdf]

Figure 1-figure supplement 1B

uncropped blots

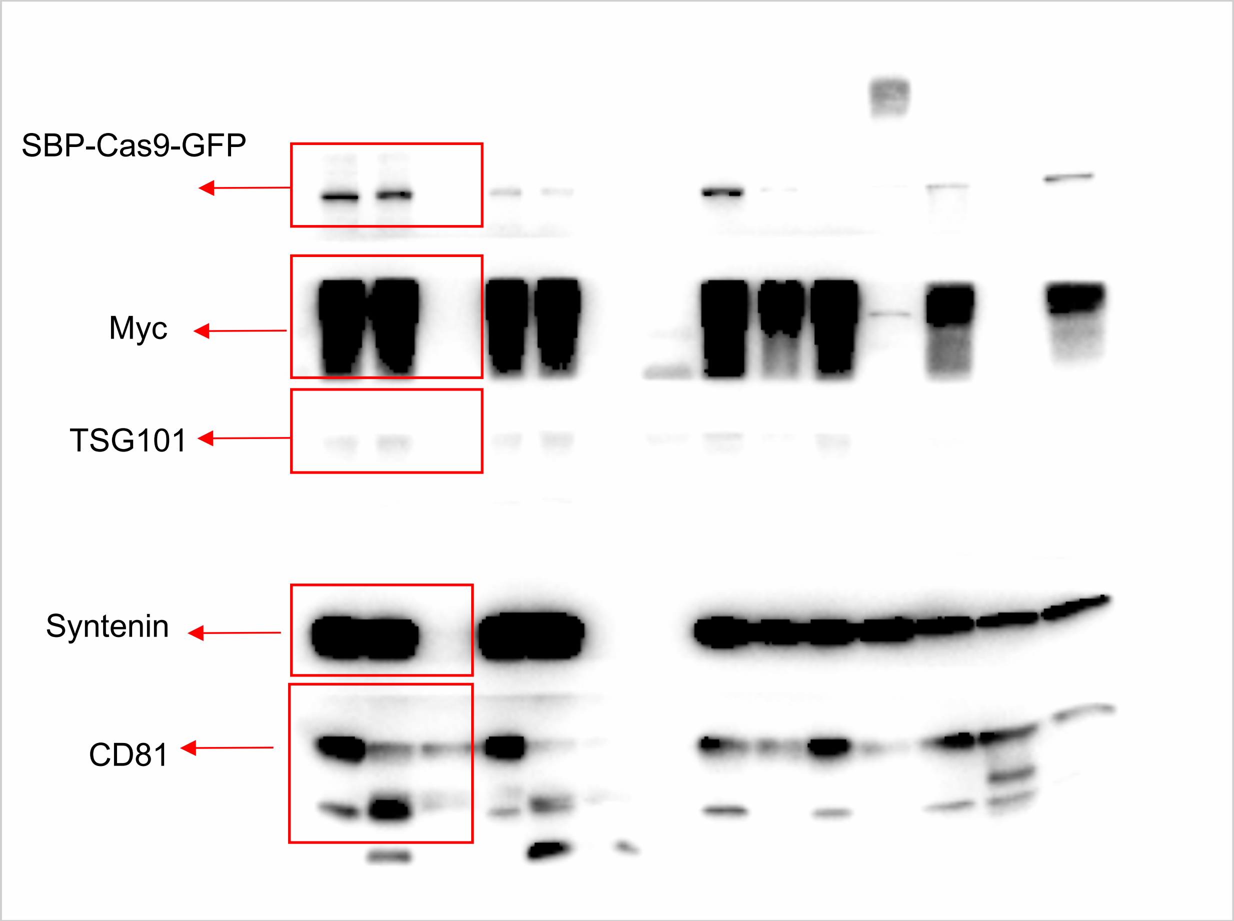

Note: the other lanes are for other experiments.

B

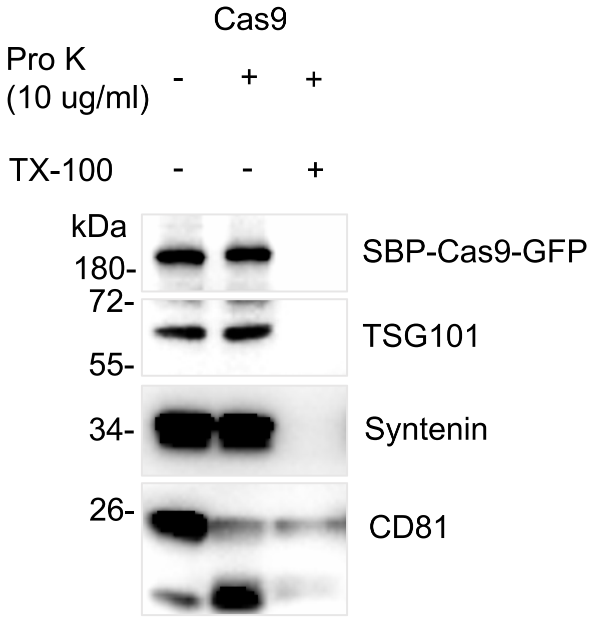

Exosomes from cells expressed both SBP-Flag-Cas9-GFP and Myc-Streptavidin-CD63-mCherry were purified and exposed to proteinase K with or without Triton X-100, showing Cas9 localized within exosomes.
