# Supplementary material for: Syncytin-mediated open-ended membrane tubular connections facilitate the intercellular transfer of cargos including Cas9 protein
Source: eLife. 2023 Mar 10;12:e84391. doi: 10.7554/eLife.84391 (PMC10112890; doi:10.7554/eLife.84391)
Supplement: Figure 3—figure supplement 2—source data 4. [file elife-84391-fig3-figsupp2-data4.zip › Figure 3-figure supplement 2-source data 4/Figure 3-figure supplement 2-source data 4.pdf]

# Figure 3-figure supplement 2D

uncropped blots

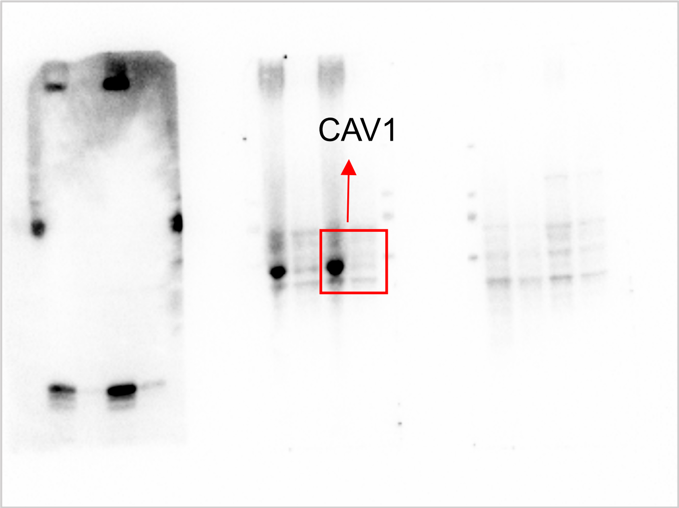


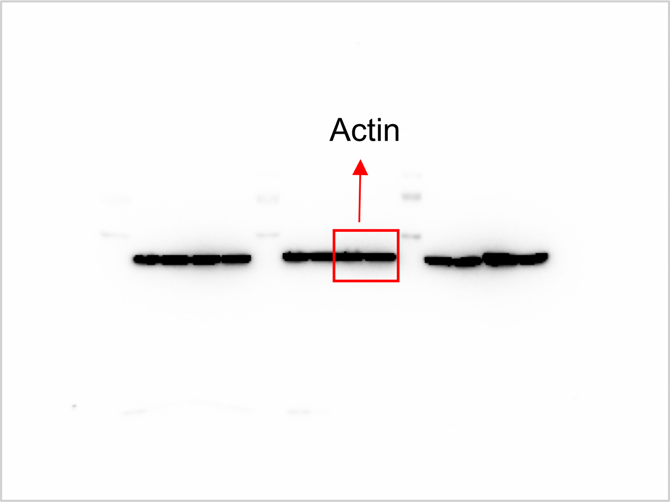


Note: the other lanes are for other experiments.

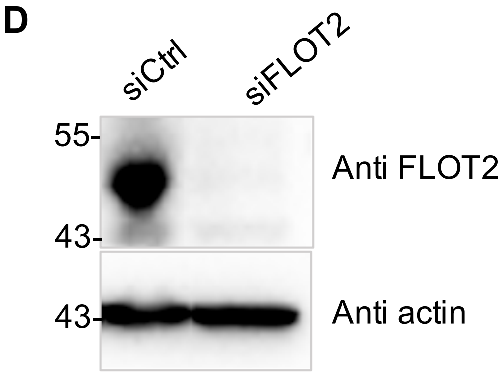


FLOT2 was knocked-down by siRNA in MDA-MB-231 cells.
