# Supplementary material for: Syncytin-mediated open-ended membrane tubular connections facilitate the intercellular transfer of cargos including Cas9 protein
Source: eLife. 2023 Mar 10;12:e84391. doi: 10.7554/eLife.84391 (PMC10112890; doi:10.7554/eLife.84391)
Supplement: Figure 3—figure supplement 2—source data 5. [file elife-84391-fig3-figsupp2-data5.zip › Figure 3-figure supplement 2-source data 5/Figure 3-figure supplement 2-source data 5.pdf]

Figure 3-figure supplement 2E, 2H

uncropped blots

Long exposure

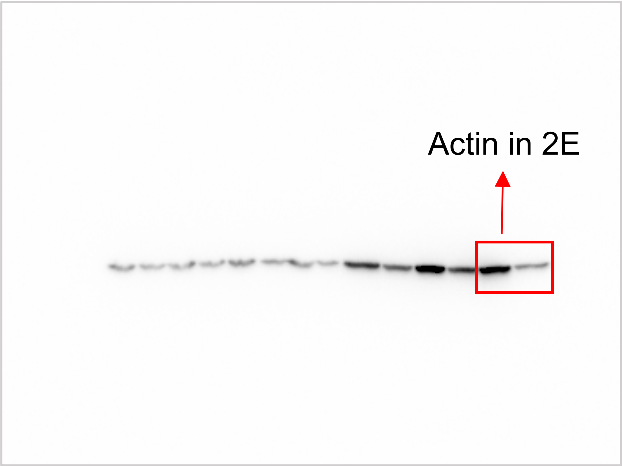

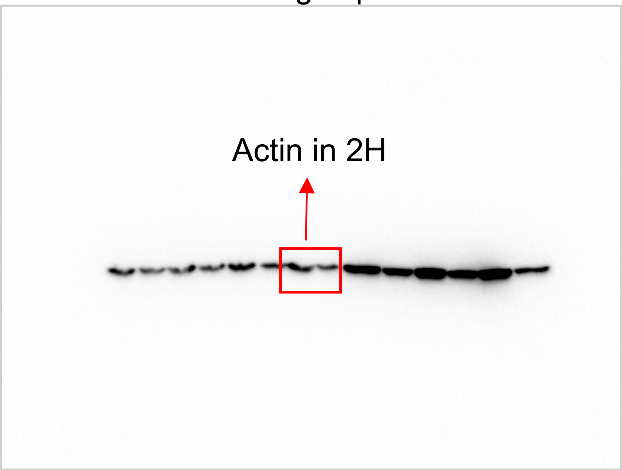

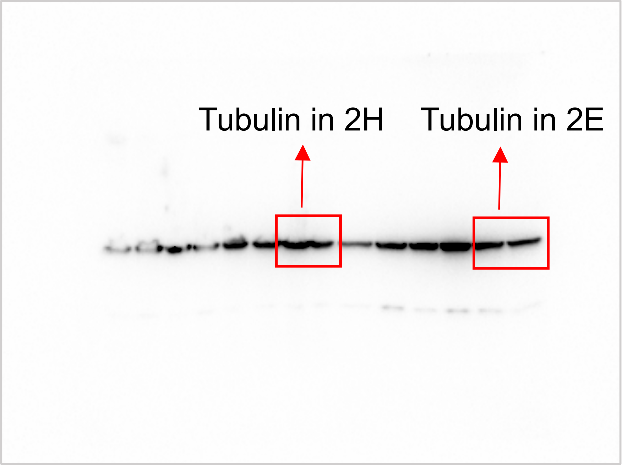

Note: the other lanes are for other experiments.

E

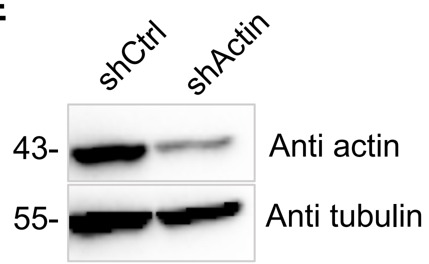

H

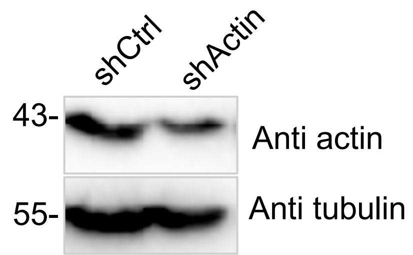

Actin was knocked-down by shRNA in MDA-MB-231 (E) or HEK293T (H) cells.
