# Supplementary material for: Syncytin-mediated open-ended membrane tubular connections facilitate the intercellular transfer of cargos including Cas9 protein
Source: eLife. 2023 Mar 10;12:e84391. doi: 10.7554/eLife.84391 (PMC10112890; doi:10.7554/eLife.84391)

Figure 3-figure supplement 2G, 2J

uncropped blots

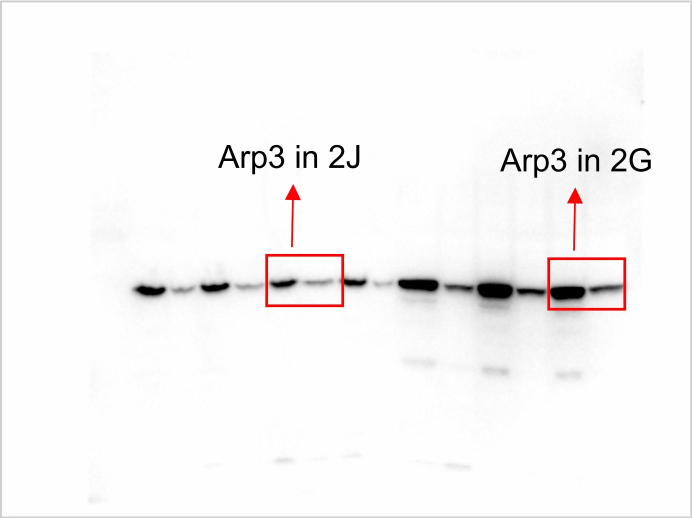

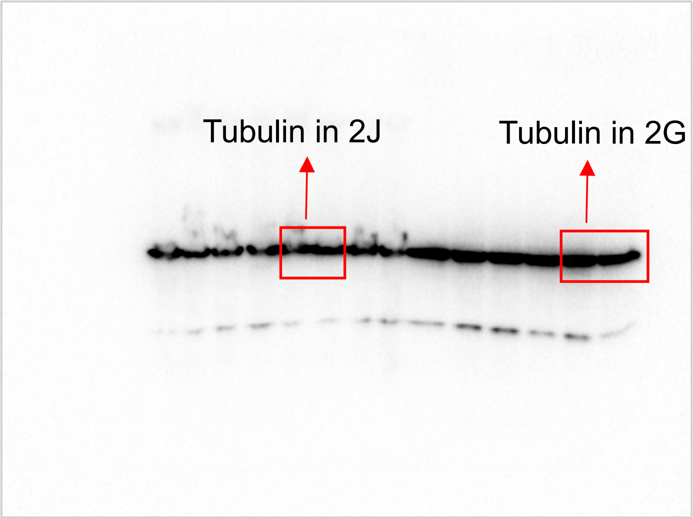

Note: the other lanes are for other experiments.

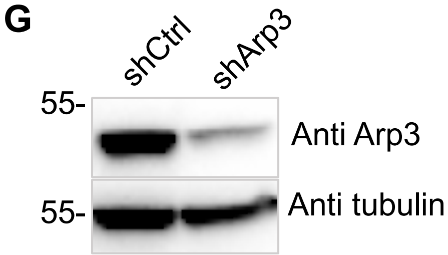

Arp3 was knocked-down by shRNA in MDA-MB-231 (G) or HEK293T (J) cells.

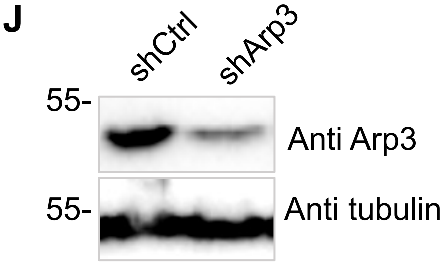

Supplement: Figure 3—figure supplement 2—source data 7. [file elife-84391-fig3-figsupp2-data7.zip › Figure 3-figure supplement 2-source data 7/Figure 3-figure supplement 2-source data 7.pdf]
