# Supplementary material for: Syncytin-mediated open-ended membrane tubular connections facilitate the intercellular transfer of cargos including Cas9 protein
Source: eLife. 2023 Mar 10;12:e84391. doi: 10.7554/eLife.84391 (PMC10112890; doi:10.7554/eLife.84391)
Supplement: Figure 7—figure supplement 1—source data 1. [file elife-84391-fig7-figsupp1-data1.zip › Figure 7-figure supplement 1-source data 1/Figure 7-figure supplement 1-source data 1.pdf]

Figure 7-figure supplement 1A

uncropped blots

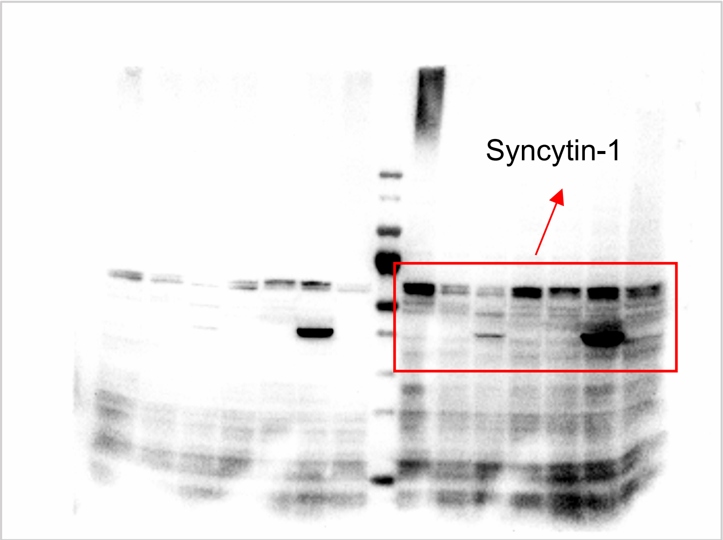

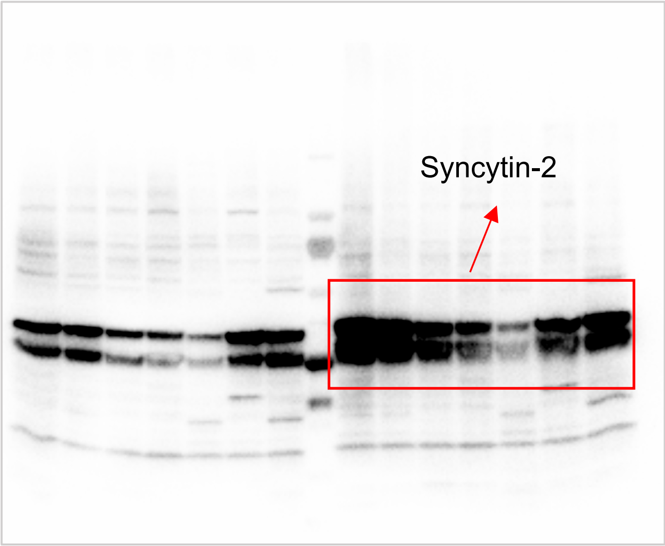

Note: the other lanes are for other experiments. The bands at other positions may be unspecific bands.

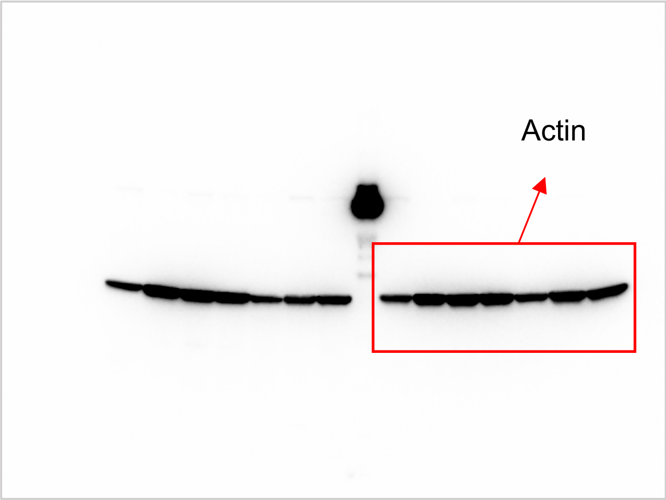

A

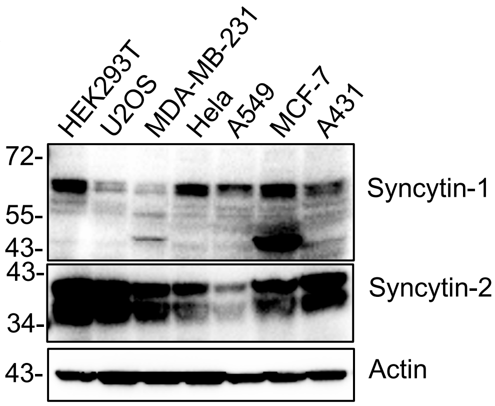

Human endogenous fusogen, syncytins expressed in different cell lines.
