# Supplementary material for: Syncytin-mediated open-ended membrane tubular connections facilitate the intercellular transfer of cargos including Cas9 protein
Source: eLife. 2023 Mar 10;12:e84391. doi: 10.7554/eLife.84391 (PMC10112890; doi:10.7554/eLife.84391)
Supplement: Figure 7—figure supplement 1—source data 2. [file elife-84391-fig7-figsupp1-data2.zip › Figure 7-figure supplement 1-source data 2/Figure 7-figure supplement 1-source data 2.pdf]

Figure 7-figure supplement 1B

uncropped blots

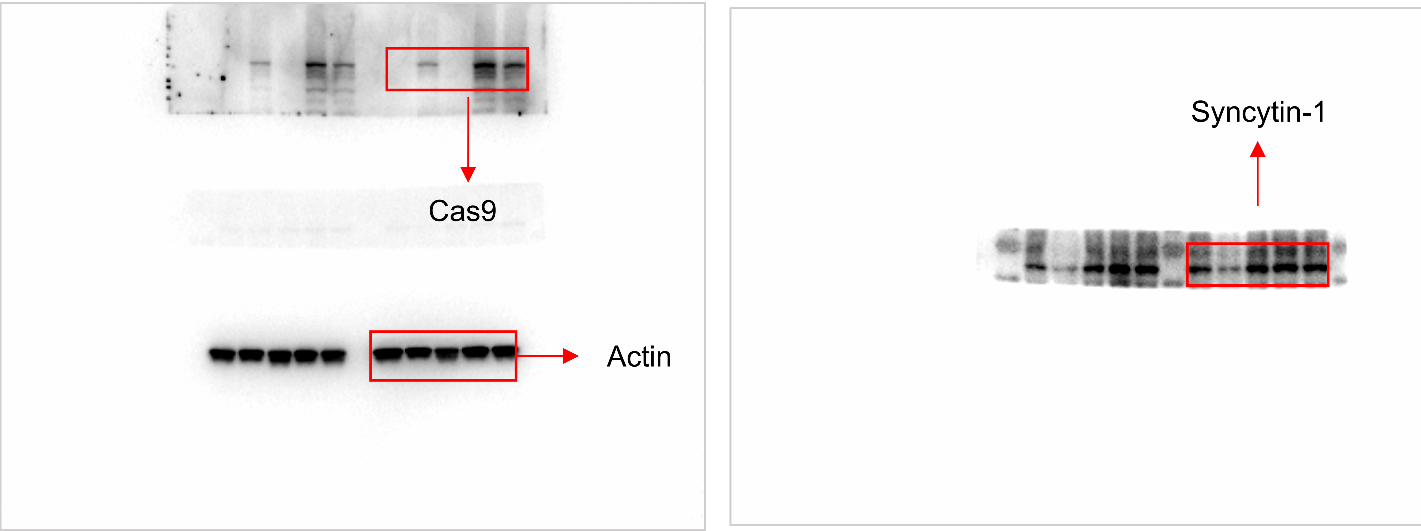

Note: the other lanes are for other experiments. The bands at other positions may be unspecific bands.

B

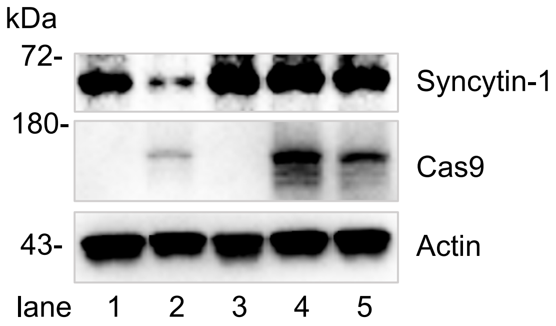

Syncytin-1 was knocked-down in MDA-MB-231 reporter cells using CRISPRi. Lane 1, reporter cell with control plasmid; lane 2, reporter cell with medium level of dCas9 and gRNA for syncytin-1 knockdown; lane 3, reporter cell with low level of dCas9 only; lane 4, reporter cell with high level of dCas9 only; lane 5, Reporter cell with medium level of dCas9 only.
