# Supplementary material for: Syncytin-mediated open-ended membrane tubular connections facilitate the intercellular transfer of cargos including Cas9 protein
Source: eLife. 2023 Mar 10;12:e84391. doi: 10.7554/eLife.84391 (PMC10112890; doi:10.7554/eLife.84391)
Supplement: Figure 7—figure supplement 1—source data 4. [file elife-84391-fig7-figsupp1-data4.zip › Figure 7-figure supplement 1-source data 4/Figure 7-figure supplement 1-source data 4.pdf]

Figure 7-figure supplement 1F

uncropped blots

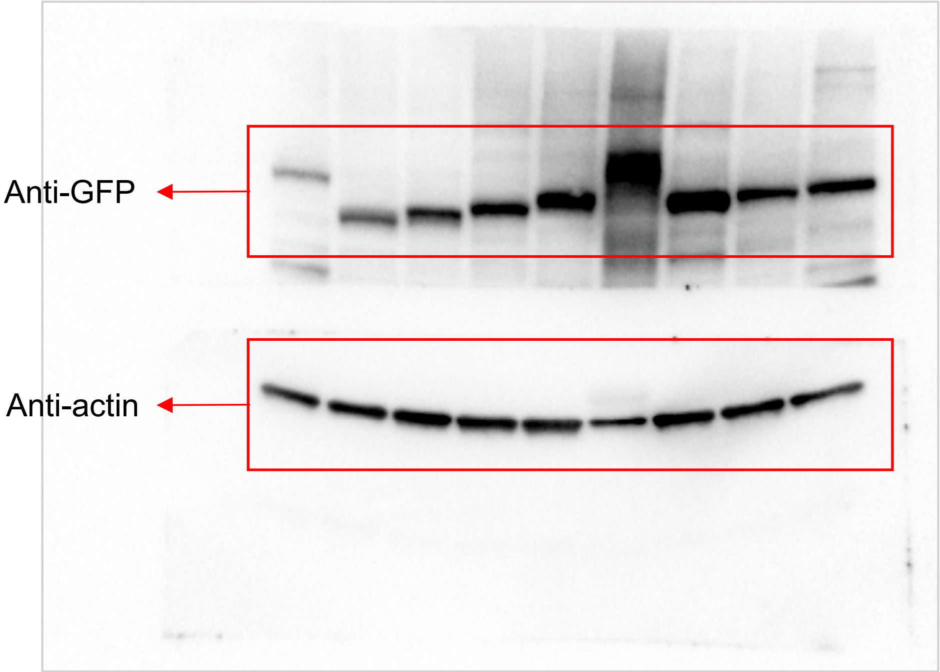

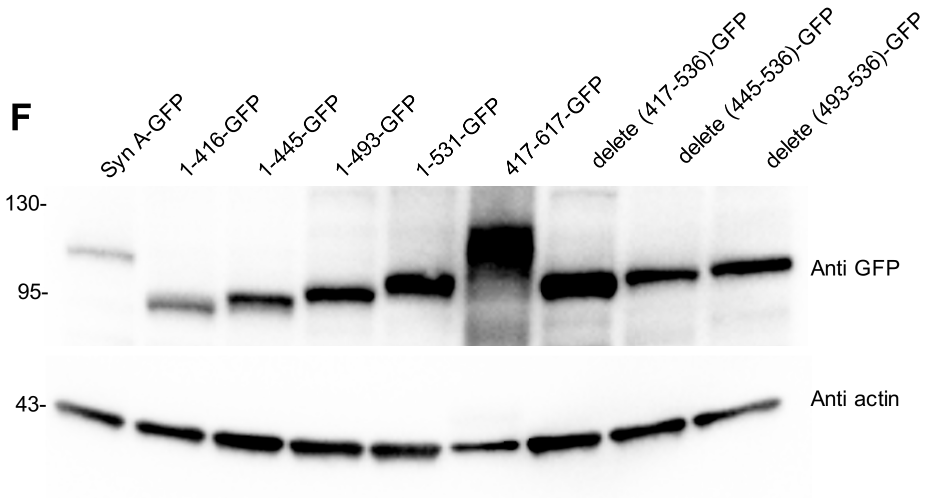

The GFP-fused syncytin-A truncations were expressed in MDA-MB-231 cells and detected by immunoblot.
