# Supplementary material for: Syncytin-mediated open-ended membrane tubular connections facilitate the intercellular transfer of cargos including Cas9 protein
Source: eLife. 2023 Mar 10;12:e84391. doi: 10.7554/eLife.84391 (PMC10112890; doi:10.7554/eLife.84391)
Supplement: Figure 9—source data 1. [file elife-84391-fig9-data1.zip › Figure 9-source data 1/Figure 9-source data 1.pdf]

Figure 9B

uncropped blots

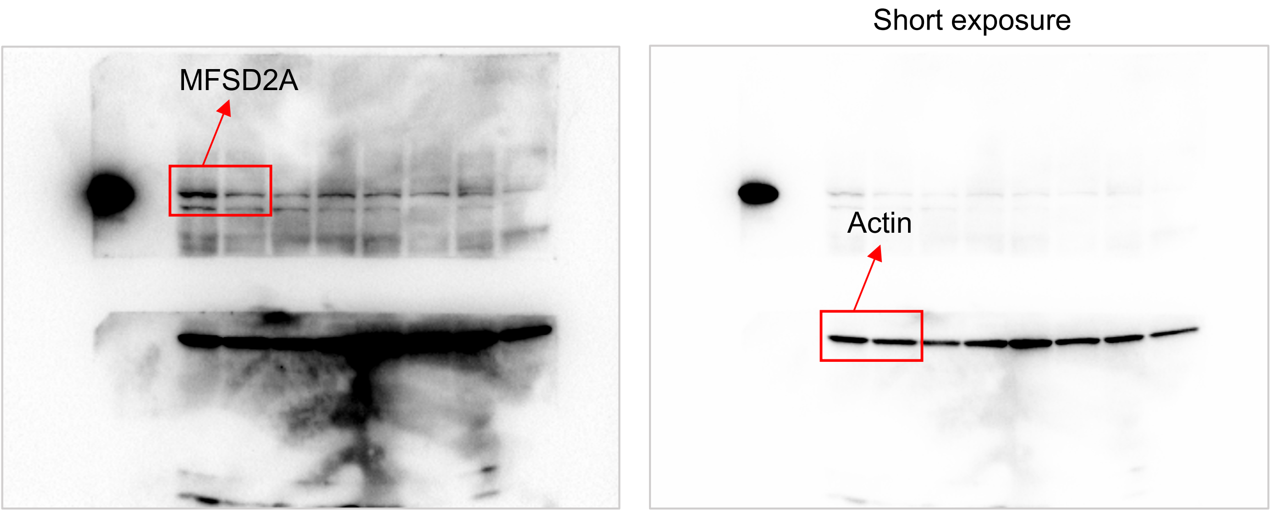

Note: the other lanes are for other experiments. The bands at other positions may be unspecific bands.

B

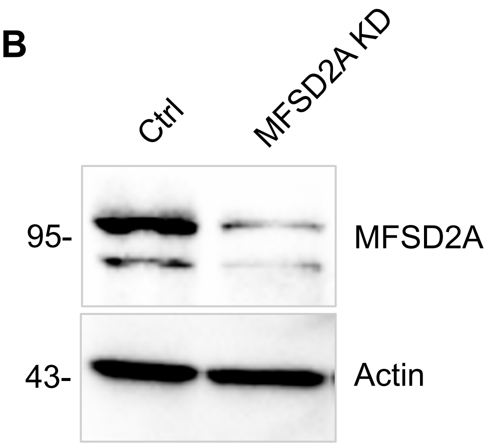

MFSD2A was knocked-down in HEK293T with stable overexpression of SBP-tagged Cas9-GFP/gRNA (SBP-Cas9/gRNA).
