# Supplementary material for: Syncytin-mediated open-ended membrane tubular connections facilitate the intercellular transfer of cargos including Cas9 protein
Source: eLife. 2023 Mar 10;12:e84391. doi: 10.7554/eLife.84391 (PMC10112890; doi:10.7554/eLife.84391)
Supplement: Figure 9—source data 2. [file elife-84391-fig9-data2.zip › Figure 9-source data 2/Figure 9-source data 2.pdf]

Figure 9D

uncropped blots

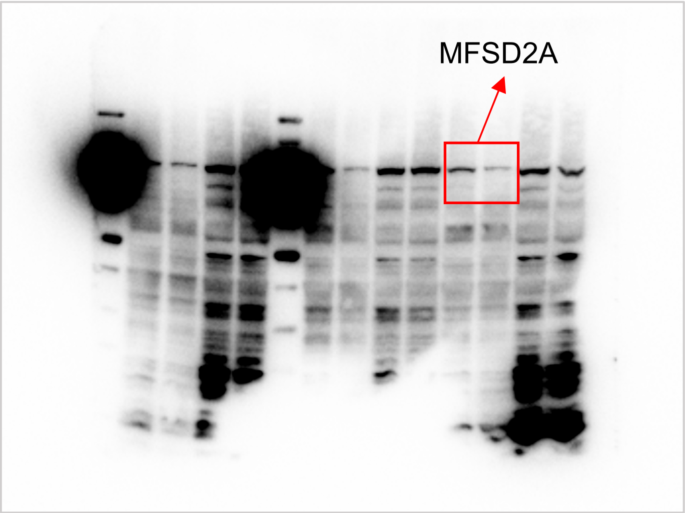

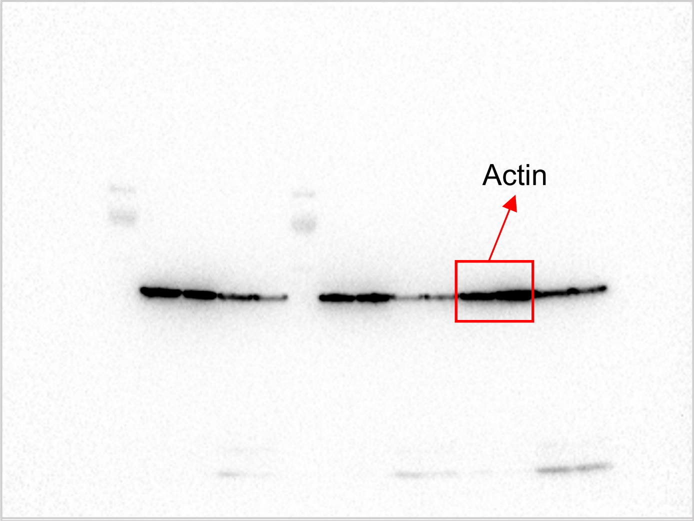

Note: the other lanes are for other experiments. The bands at other positions may be unspecific bands.

D

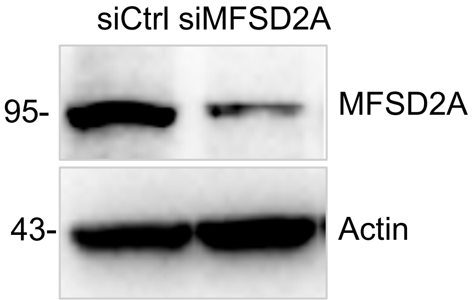

MFSD2A was knocked-down in MDA-MB-231 with reporter plasmid.
